# Starch phosphorylase acts as a pivotal scaffold connecting starch metabolism enzymes with photosystem I subunits

**DOI:** 10.1093/pcp/pcag063

**Published:** 2026-05-14

**Authors:** Seon-Kap Hwang, Li Tian, Chun-Yeung Ng, Thomas W Okita

**Affiliations:** Institute of Biological Chemistry, Washington State University, Pullman, WA 99164-7411, USA; Institute of Biological Chemistry, Washington State University, Pullman, WA 99164-7411, USA; Key Laboratory of Quality and Safety Control for Subtropical Fruit and Vegetable, Ministry of Agriculture and Rural Affairs, Collaborative Innovation Center for Efficient and Green Production of Agriculture in Mountainous Areas of Zhejiang Province, College of Horticulture Science, Zhejiang A&F University, Hangzhou, Zhejiang, China; Institute of Biological Chemistry, Washington State University, Pullman, WA 99164-7411, USA; Institute of Biological Chemistry, Washington State University, Pullman, WA 99164-7411, USA

**Keywords:** starch metabolism, photosystem I, starch phosphorylase, protein–protein interaction, PsaC and PsaD, heterocomplex, CRISPR technology

## Abstract

In a previous study, we proposed that starch phosphorylase (Pho1) interacts with PsaC, a core subunit of photosystem I (PSI), although it was not clear whether the interaction was a direct 1:1 relationship or by indirect association with another factor. Here, we validated the Pho1–PsaC interaction in vivo using bimolecular fluorescence complementation analysis. Pulldown assays with wild-type, BMF136, and the Pho1-null plants revealed that glutathione S-transferase-Pho1 consistently associates with PsaC and the starch disproportionating enzyme (Dpe1) across all genotypes. These results indicate that Pho1 is essential for forming a multi-protein complex containing PsaC and Dpe1. Yeast 2-hybrid assays further validated the direct Pho1–PsaC interaction and also demonstrated that Pho1 interacts with PsaD, another subunit of PSI, but not with PsaE, indicating that PsaD functions as an additional component of the Pho1-associated heterocomplex. PsaC bound poorly to the Pho1 catalytically null variants, although PsaC fused to a HaloTag restored interaction with the catalytic null mutants, implying that the HaloTag facilitated or stabilized complex formation. Our results indicate that Pho1 is an essential factor linking starch biosynthesis to photosynthesis through its ability to form multi-protein complexes with starch metabolism enzymes and PSI subunits.

## Introduction

Starch, a polysaccharide composed of amylose and amylopectin, serves as the primary carbohydrate reserve in many plant tissues, including seeds, tubers, and leaves. The synthesis of starch occurs primarily in plastids and involves several enzymatic steps that convert glucose into amylose and amylopectin, the two main components of starch. Starch biosynthesis entails a complex process involving multiple enzyme activities and their isoforms. These enzymes include those considered to have a primarily degradative function. One enzyme that serves in both biosynthetic and degradative activities is starch phosphorylase (Pho1) (Hwang et al. 2020, Seung et al. 2025). This enzyme activity exists in two isoforms, Pho1 and Pho2, which differ in their catalytic activities, regulatory mechanisms, and subcellular locations (Cuesta-Seijo et al. 2017, Nakamura et al. 2022), allowing plants to fine-tune their starch metabolism in response to developmental and environmental cues. Starch Pho1 catalyzes a reversible reaction, Glc 1P + *α*-glucan_(*n*)_ ↔ P*_i_* + *α*-glucan_(*n* + 1)_ (Hwang et al. 2010, 2016a), which depends on the developmental clock and the type of plant tissues. This enzyme plays a crucial role in starch metabolism by mobilizing stored energy, particularly during seed germination, and by starch biosynthesis during plant growth and seed development. The regulation of Pho1 is tightly controlled by various factors, including the availability of substrates, energy status, and hormonal signals (Fulton et al. 2008, Shoaib et al. 2021).

As a starch biosynthetic enzyme, Pho1 functionally interacts with branching enzymes, specifically starch branching enzyme I (BEI), IIa (BEIIa), and/or IIb (BEIIb) (Crofts et al. 2015, Nakamura et al. 2017, 2012, Tetlow et al. 2004). These interactions are crucial for the coordinated regulation of starch biosynthesis and degradation. Studies have shown that several of these enzymes co-immunoprecipitate, indicating that they physically interact stably within plant cells, particularly when subjected to protein phosphorylation conditions in amyloplasts (Tetlow et al. 2004, Liu et al. 2009). We had previously observed that Pho1 also interacts with disproportionating enzyme (also called Dpe1 or D-enzyme), where formation of the enzyme complex appears to enhance the utilization of α-glucans in plant cells (Hwang et al. 2016b). The formation of such complexes suggests a functional collaboration where starch Pho1 and Dpe1 generate linear maltodextrins that can subsequently be acted upon by branching enzymes to create branched structures in starch (Hwang et al. 2016a). Other proteins were also identified to interact with Pho1 to regulate plant metabolism (Tetlow et al. 2004, Hennen-Bierwagen et al. 2009, Crofts et al. 2015, Koper et al. 2021, Muntaha and Fettke 2025).

Comparative analysis of glucan phosphorylases across various organisms revealed that starch phosphorylases (Pho1s) from higher plants exclusively contain an intrinsically disordered domain, comprising about 50–82 amino acids depending on the plant species (Nakano and Fukui 1986, Hwang et al. 2020, Yu et al. 2022). This domain is not essential for the enzyme’s catalytic properties but confers partial enzyme stability at high temperature (Hwang et al. 2016a). When the L80 region was deleted from rice Pho1 and the modified enzyme expressed in the rice Pho1 knockdown mutant BMF136, the transgenic plants showed considerable improvements in agronomic traits, including faster growth rate, early flowering, increased biomass, and larger grains and grain yields. These performance gains suggest that the L80 domain may act as a negative regulator of Pho1 function (Koper et al. 2021). Additionally, these transgenic plants exhibited altered photosynthetic traits: i.e. enhanced carbon assimilation and modified electron flow through PSI. Further studies using pulldown assays and co-immunoprecipitation indicated that Pho1 interacts with PsaC, a subunit of PSI located in the thylakoid membranes of chloroplasts (Koper et al. 2021). PSI plays an essential role in converting light energy into chemical energy by transferring electrons from plastocyanin to ferredoxin, a key step in the production of reduced nicotinamide adenine dinucleotide phosphate (NADPH)—an important reducing agent in the Calvin cycle (Fromme et al. 2003, Jensen et al. 2007, Naschberger et al. 2024). On the stromal side of PSI, PsaC, PsaD, and PsaE form a multiprotein complex involved in electron transfer (Fromme et al. 2003, Amunts et al. 2010, Naschberger et al. 2024). PsaC is an apoprotein with two [4Fe-4S] clusters (FA and FB), which are integral for electron transfer. PsaD assists in the interaction with ferredoxin, while PsaE, positioned adjacent to both PsaC and PsaD, facilitates electron transfer by providing a docking site for ferredoxin on the stromal surface of the complex. Of the three subunits, only PsaC is encoded by plastid DNA.

While these findings suggest an unconventional yet direct interaction between Pho1 and the PSI complex, further evidence is required to confirm this relationship. In this study, we employed bimolecular fluorescence complementation (BiFC), pull-down, and yeast 2-hybrid (Y2H) assays to investigate in-depth the interaction between Pho1 and PsaC. Furthermore, we investigated whether other plastidial proteins, including Dpe1 and additional PSI subunits, also interact with Pho1 and PsaC.

## Results

### Phosphorylase interacts with PsaC in BY-2 cells

**Figure 1:**
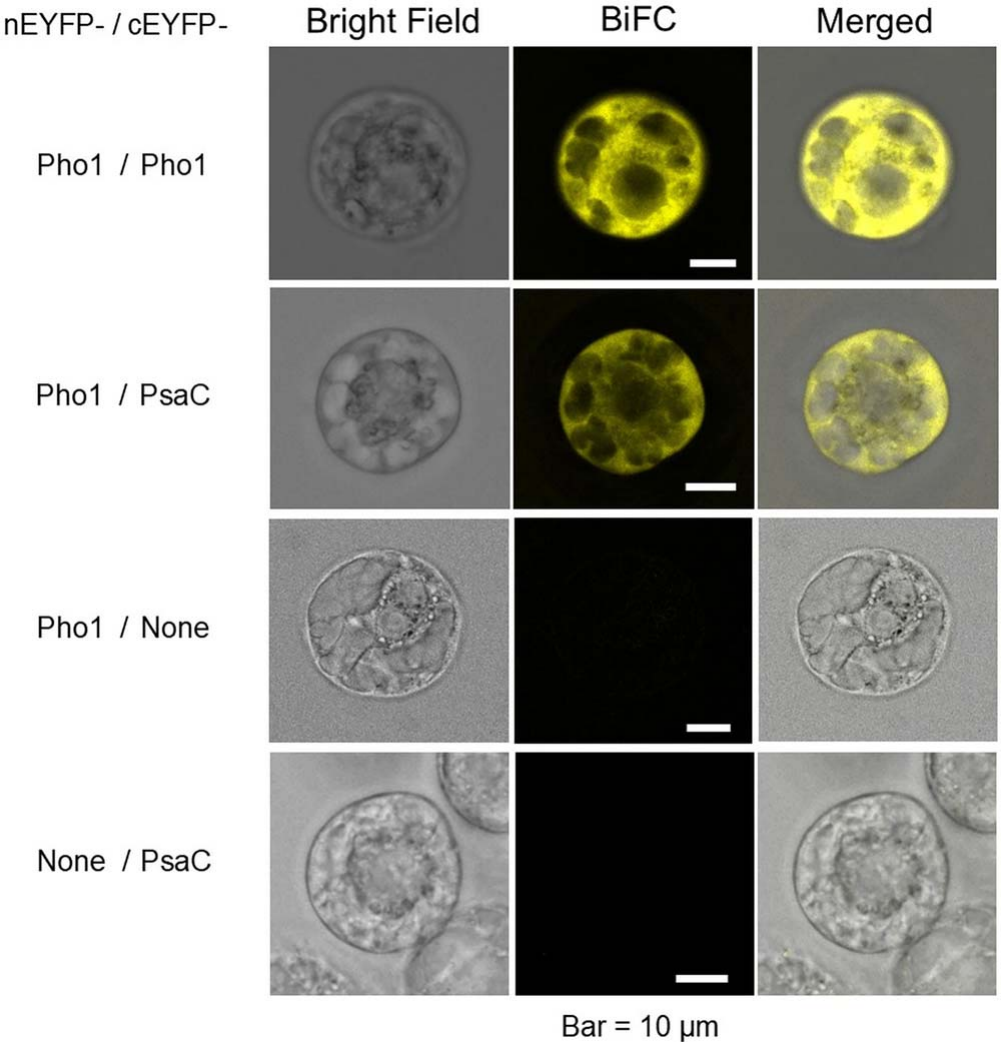
BiFC analysis of the interaction between Pho1 and PsaC in *Nicotiana tabacum* BY-2 cells. Constructs encoding Pho1 fused to the N-terminal half of EYFP (nEYFP-Pho1) and Pho1 or PsaC fused to the C-terminal half of EYFP (cEYFP-Pho1 or cEYFP-PsaC) were transiently expressed in BY-2 cells after PEG-mediated transformation. nEYFP-Pho1/cEYFP and nEYFP/cEYFP-PsaC were used as negative controls. YFP fluorescence was visualized by confocal microscopy 16 h after transformation. Left panels: brightfield images of the same cells; middle panels: YFP signal observed in the cytoplasm; right panels: merged image of YFP fluorescence and brightfield to confirm subcellular localization. “None” indicates empty vectors expressing nEYFP and cEYFP alone. Scale bars = 10 *μ*m.

To investigate the interaction in living cells between the rice Pho1 enzyme and PsaC, a subunit of the photosystem I (PSI) complex, we performed a BiFC assay. As a positive control, the Pho1 constructs fused to the N-terminal enhanced yellow fluorescent protein-Pho1 (nEYFP-Pho1) and C-terminal EYFP-Pho1 (cEYFP-Pho1) fragments of EYFP were co-expressed in tobacco BY-2 cells. Clear reconstitution of YFP fluorescence between Pho1 was observed (Fig. 1, first row), indicating a physical interaction between the two Pho1 proteins, whose normal quaternary structure is a pair of subunits (Hwang et al. 2016b, Cuesta-Seijo et al. 2017). When nEYFP-Pho1 and cEYFP-PsaC were co-expressed, we also observed distinct, albeit weaker, YFP fluorescence, confirming a physical interaction between Pho1 and PsaC (Fig. 1, second row). Notably, this fluorescence signal was localized predominantly in the cytosolic space of the protoplasts, consistent with the fact that a mature (transit peptide-less) form of Pho1 was used and PsaC naturally lacks a chloroplast transit peptide. In contrast, negative control combinations—nYFP-Pho1 co-expressed with cEYFP alone (Fig. 1, third row), and nEYFP co-expressed with cEYFP-PsaC (Fig. 1, bottom row)—did not produce detectable fluorescence, ruling out spontaneous reconstitution of the fluorophore and confirming the specificity of the Pho1–PsaC interaction. These findings reinforce our previous data and support the hypothesis that Pho1 can associate with PSI by interacting with PsaC (Koper et al. 2021).

### Generation of phosphorylase null mutant rice

To generate a Pho1 knockout (null) mutant completely lacking Pho1 protein expression, we employed Clustered Regularly Interspaced Short Palindromic Repeats (CRISPR)-Cpf1 genome editing technology (Tang et al. 2017). A guide RNA was designed to specifically target exon-2 of the Pho1 coding sequence, a region critical for the structural and functional integrity of the enzyme. *Agrobacterium tumefaciens*-mediated transformation using pSH984 generated over 50 independent T_0_ transgenic plants. Transgenic plants were screened by polymerase chain reaction (PCR) amplification of the genomic region flanking the target site in exon-2, and the PCR products were sequenced to identify insertions or deletions (indels) that disrupted the reading frame and resulted in loss-of-function alleles. We identified six homozygous T_2_ lines (two progenies each of #24, #72, and #92) carrying frameshift mutations in the *PHO1* gene (Supplementary Fig. S1a). Lines #24, #72, and #92 contain nucleotide deletions of 28, 8, and 19 bp, respectively, within exon 2 of the gene, which are predicted to cause premature termination of the Pho1 protein and the loss of intact Pho1 protein and formation of truncated proteins 12.1 kDa or smaller (Supplementary Fig. S1a). To confirm the loss of intact Pho1 protein expression, total protein extracts from mature T2 seeds of homozygous plants were analyzed by immunoblotting with a Pho1-specific polyclonal antibody. The CRISPR-generated knock-out lines showed no detectable full-length or truncated Pho1 proteins 25–30 kDa or larger (Supplementary Fig. S1b). The absence of Pho1 signals larger than 30 kDa is consistent with premature termination of translation. These null mutants differed from the BMF136 mutant, which expresses very low levels of both the wildtype Pho1 protein and a slightly smaller Pho1 variant carrying an internal 53-amino acid deletion (Supplementary Fig. S1b; Supplementary Table S1), a change that appears to significantly weaken protein–protein interactions between Pho1 subunits (Supplementary Table S1).

### Pho1, Dpe1, and PsaC form multimeric complexes

**Figure 2:**
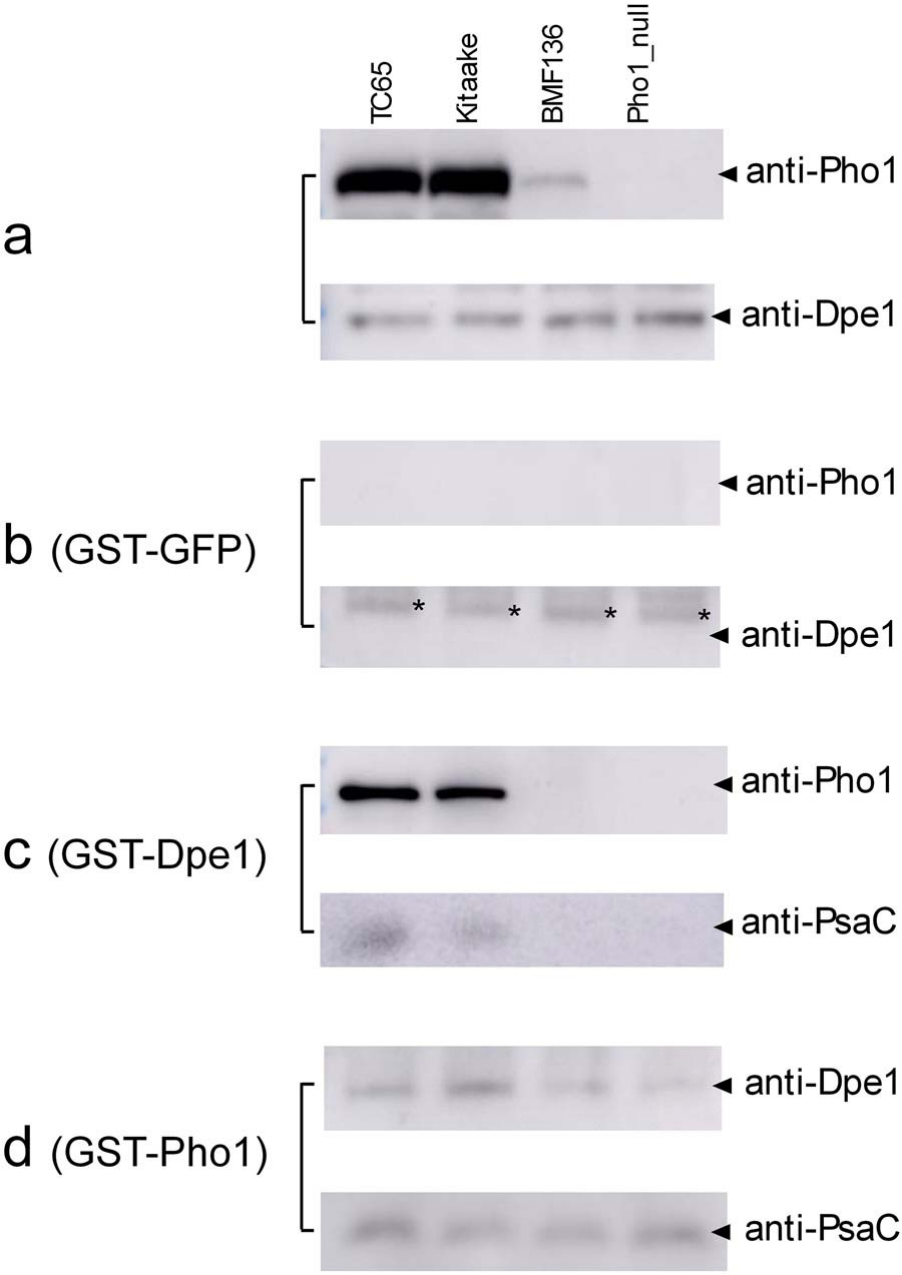
Pulldown assays of Pho1, Dpe1, and PsaC in rice wildtype (cv. TC65 and cv. Kitaake), BMF136, and Pho1 knockout (null) transgenic plants. Soluble proteins were extracted from whole immature seeds (7 DAF), including the pericarp and endosperm tissues, and screened using antibodies raised against purified rice Pho1 or Dpe1. A commercially available PsaC antibody (PytoAB), raised against a conserved peptide sequence from *Arabidopsis thaliana* PsaC, was used. (a) Soluble seed extract (before pulldown); (b) pulldown using GST-GFP (negative control); (c) pulldown using GST-Dpe1; (d) pulldown using GST-Pho1. Pho1 and Dpe1 signals were detected using a SuperSignal West Pico Plus Chemiluminescence kit, whereas PsaC signals were detected with a SuperSignal West Femto Chemiluminescence kit due to the low abundance of PsaC in the eluates. Asterisks denote non-specific bands eluted from the GST-GFP pulldown (negative control), which are 4–5 kDa larger than Dpe1. Arrowhead indicates the expected molecular mass of each respective protein.

To examine potential protein–protein interactions among Pho1, Dpe1, and PsaC in developing rice seeds, we performed glutathione S-transferase (GST) pull-down assays using protein extracts from two wild-type rice cultivars (TC65 and Kitaake) as well as the Pho1 knockdown (BMF136) and Pho1 knockout (null; #72–3) mutant lines (Fig. 2). Two wildtype cultivars were included because BMF136 was derived from TC65, while the null mutants originated from Kitaake. When total soluble proteins were probed with Pho1-specific antibodies, strong Pho1 signals were detected in both wildtype cultivars, whereas BMF136 exhibited only very low levels while the null mutant showed no detectable Pho1 (Fig. 2a), consistent with Supplementary Fig. S1. Immunoblotting with a Dpe1-specific antibody showed comparable Dpe1 expression across all samples (Fig. 2a). Soluble protein extracts were incubated with glutathione-agarose resin pre-bound with GST-GFP, GST-Pho1, or GST-Dpe1 fusion proteins. After extensive washing to remove non-specifically bound proteins, the proteins were eluted, resolved by sodium dodecyl sulfate-polyacrylamide gel electrophoresis (SDS-PAGE), and analyzed by immunoblotting with Pho1-, Dpe1-, and PsaC-specific antibodies. No Pho1 or Dpe1 signals were observed in eluates from the GST-GFP controls (Fig. 2b), confirming the absence of non-specific protein binding. GST-Dpe1 pull-downs probed with Pho1- and PsaC-specific antibodies revealed the presence of both Pho1 and PsaC in eluates from wild-type TC65 and Kitaake, but not from BMF136 or #72–3 (Fig. 2c), consistent with the known Pho1 deficiency in these mutant lines. These results indicate that GST-Dpe1 can capture Pho1 only when sufficient levels of Pho1 are present. However, GST-Pho1 effectively pulled down both Dpe1 and PsaC across all samples (Fig. 2d), suggesting that Pho1 can directly or simultaneously associate with both proteins. Collectively, these findings raise the possibility that Pho1 forms a heteromeric complex with Dpe1 and PsaC in wildtype rice and may function as a molecular scaffold or stabilizer that facilitates Dpe1–PsaC interaction.

### Disproportionating enzyme does not interact with the L80 region of phosphorylase

**Figure 3:**
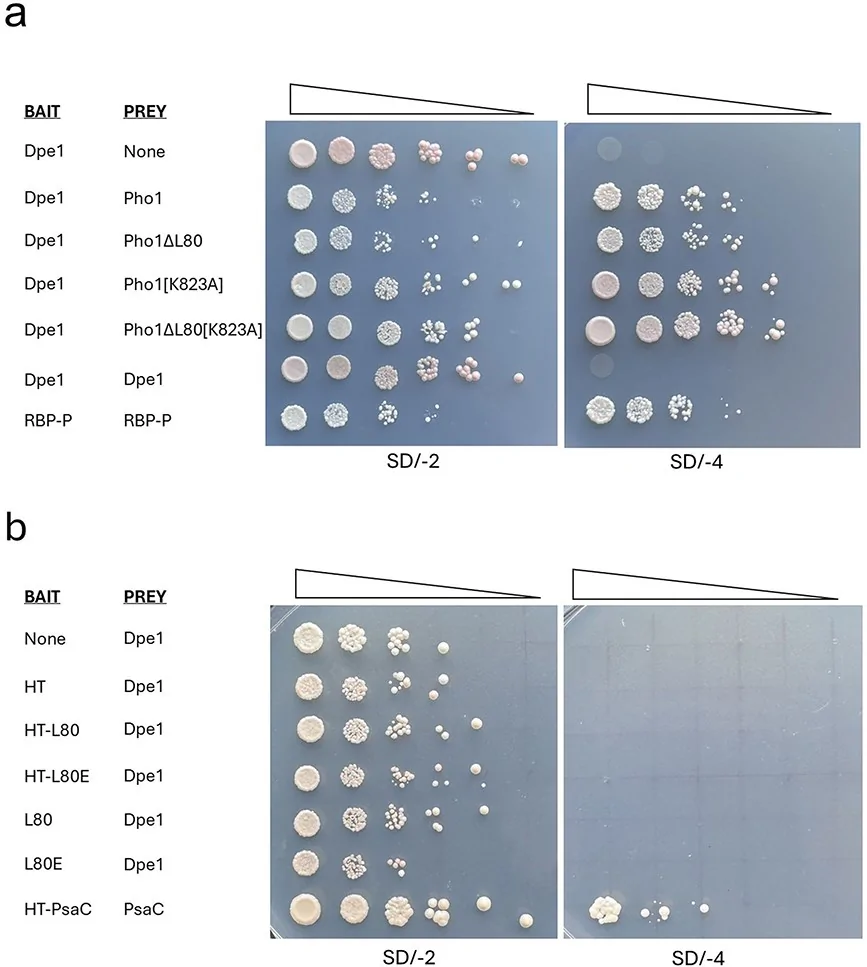
Y2H assay examining interactions of Dpe1 with Pho1 variants and L80 peptides. Yeast strain AH109 was co-transformed with plasmids encoding bait proteins (Dpe1, L80, and L80E) fused to the GAL4 DNA-binding domain (GBD) and prey proteins (Dpe1 and Pho1 variants) fused to the GAL4 activation domain (GAD). Plasmid names and schematic maps of the expressed proteins are shown in Supplementary Fig. S2. Transformants were selected on SD/−Leu/−Trp medium (SD/−2) and serially diluted (1:10) before being spotted onto the same selective medium and onto SD/−Leu/−Trp/−His/−Ade medium (SD/−4) to assess interaction-dependent reporter gene activation. Growth on selective plates indicates a positive protein–protein interaction. Co-expression of Dpe1 with empty vector pGBKT7.1 or pGADT7.1 (none) served as negative controls, while co-expression of BRP-P proteins (Tian et al. 2018) was used as a positive control. (a) Protein–protein interactions between Dpe1 and various Pho1 variants. (b) Interactions between L80 constructs (L80, L80E, HT-L80, and HT-L80E) and Dpe1.

Previous studies have observed interactions of Pho1-Dpe1 and Pho1∆L80-Dpe1 (Hwang et al. 2016b). To determine whether a catalytic mutation impacts the interactions, we performed Y2H assays. We co-transformed plasmid DNAs expressing GBD-bait and GAD-prey proteins into AH109 cells, and the colonies were grown both in SD/−2 and SD/−4 plates. Consistent with our earlier results, Fig. 3a shows that Dpe1 interacts similarly with both Pho1 and Pho1∆L80. Moreover, Dpe1 also displayed strong interactions with the catalytic mutants of Pho1 and Pho1∆L80, in which lysine-823, the pyridoxal 5′-phosphate (PLP) binding residue, was replaced with alanine. This substitution rendered the enzymes (Pho1[K823A] and Pho1ΔL80[K823A]) catalytically inactive. This result indicates that the L80 and K823A mutations do not disrupt the interaction with Dpe1. However, when the L80 domain and an extended region containing this domain were tested for interaction with Dpe1, no interactions were observed (Fig. 3b). This suggests that the L80 domain and its neighboring regions in Pho1 are not involved in protein–protein interaction with Dpe1.

### Phosphorylase interacts with PsaC and PsaD by yeast 2-hybrid

**Figure 4:**
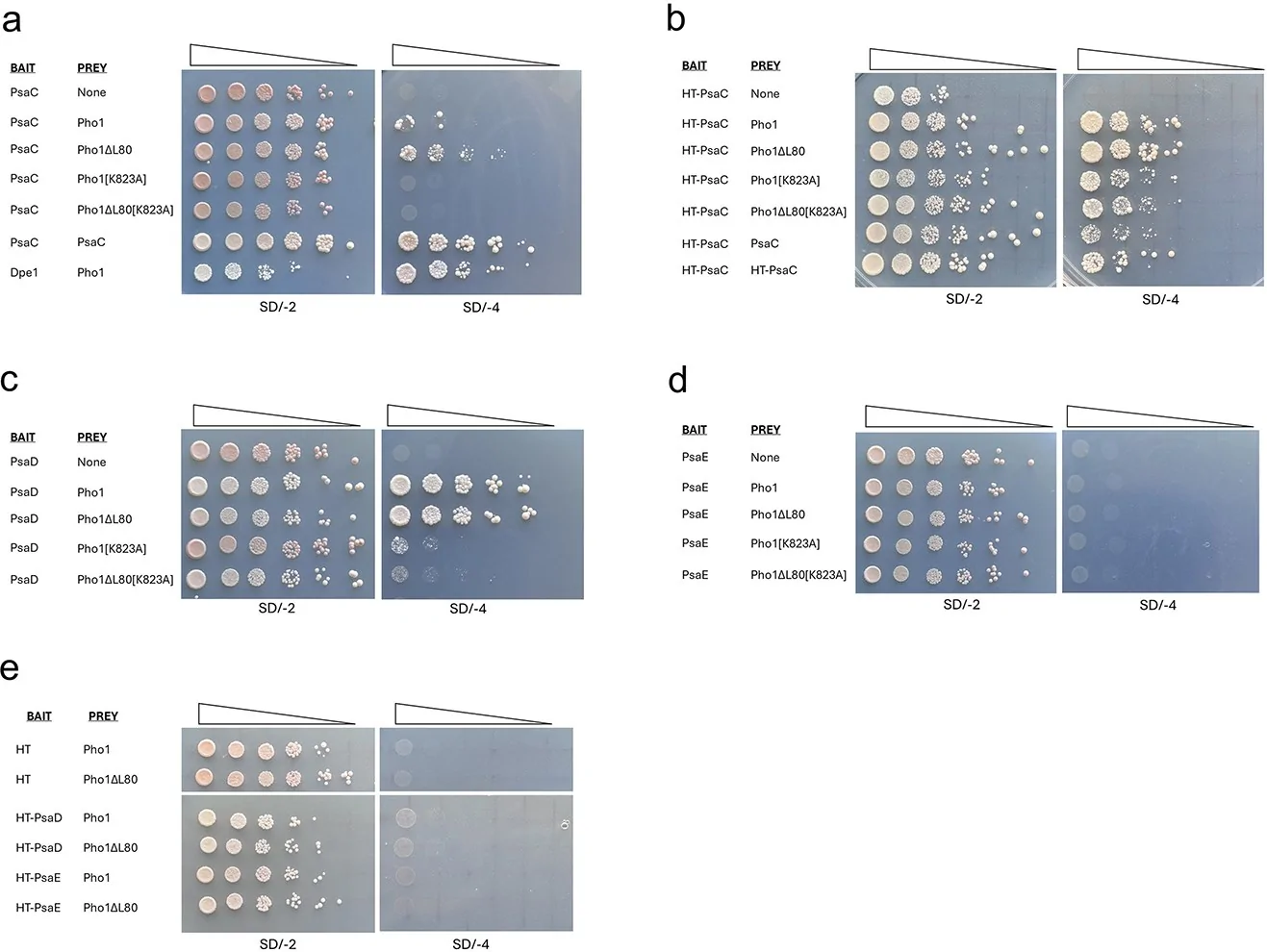
Y2H assay showing interaction between PSI subunits and Pho1 variants. Yeast strain AH109 was co-transformed with plasmids encoding bait proteins fused to GBD and prey proteins fused to GAD. Plasmid names and schematic maps of the expressed proteins are shown in Supplementary Fig. S2. Co-expression of pGBKT7.1 or pGADT7.1 empty vector (None) served as negative controls, and co-expression of Dpe1 and Pho1 proteins was used as positive controls. Protein–protein interactions were tested between Pho1 variants and (a) PsaC, (b) PsaD, (c) PsaE, (d) HT-PsaC, (e) HT-PsaD and HT-PsaE.

As a control, only GBD or GAD was transformed with either PsaC or Pho1. The result showed that PsaC+Pho1 formed colonies on both media, while the controls only grew on SD/-2 alone, indicating an interaction between PsaC and Pho1. We extended our studies to examine whether a deletion of L80 affects the interaction between two proteins. Based on Y2H analysis, PsaC interacts somewhat better with Pho1∆L80 than with wild-type Pho1 (Fig. 4a).

We examined whether the K823A catalytic mutation affects its interactions with other proteins. Based on Y2H analysis, PsaC showed no significant interaction with either of the catalytic-deficient forms (Pho1[K823A] and Pho1∆L80[K823A]) (Fig. 4a), suggesting lysine-823 is important for PsaC–Pho1 interaction. However, when PsaC was fused with a HaloTag, the resulting HaloTag–PsaC was able to interact with the Pho1 catalytic null forms in addition to Pho1 and Pho1∆L80 (Fig. 4b). We further examined the interaction of Pho1 with additional PSI subunits, PsaD and PsaE (Fig. 4c and d). When PsaD was used for protein–protein interaction analysis, the results were very similar to those obtained with PsaC. PsaD interacted with Pho1 and Pho1∆L80 (Fig. 4c) with an interaction much stronger than with PsaC in yeast cells (Fig. 4a). Similar to PsaC, PsaD interacted very poorly with the catalytic null enzyme forms (Fig. 4c), indicating the K823 residue of the enzyme is essential for Pho1-PsaD interaction. Interestingly, when PsaD was fused to HaloTag, the resulting HaloTag–PsaD lost its ability to interact with Pho1 and Pho1∆L80 (Fig. 4e), indicating the HaloTag fusion interferes with PsaD binding to Pho1 and Pho1∆L80. Neither PsaE nor HaloTag–PsaE interacted with Pho1 and Pho1 variants (Fig. 4d and e).

### L80 domain does not interact with PsaC, PsaD, and PsaE

We conducted Y2H assays to examine whether the L80 domain—an independent structural domain of Pho1—interacts with PSI subunits located on the stromal side of chloroplast. Both the isolated L80 peptide and an extended version containing its N- and C-terminal flanking regions (L80E) (Supplementary Fig. S2) were tested as preys against bait proteins PsaC, HaloTag-PsaC, PsaD, and PsaE in yeast cells. The results showed that yeast cells co-expressing the bait proteins with either L80 construct did not grow on SD/−4 medium (Supplementary Fig. S3). Reciprocal assays yielded the same results, indicating that the L80 domain does not interact with PSI subunits PsaC, PsaD, and PsaE.

### Interaction specificity of photosystem I stromal subunits and effects of HaloTag tagging

**Figure 5:**
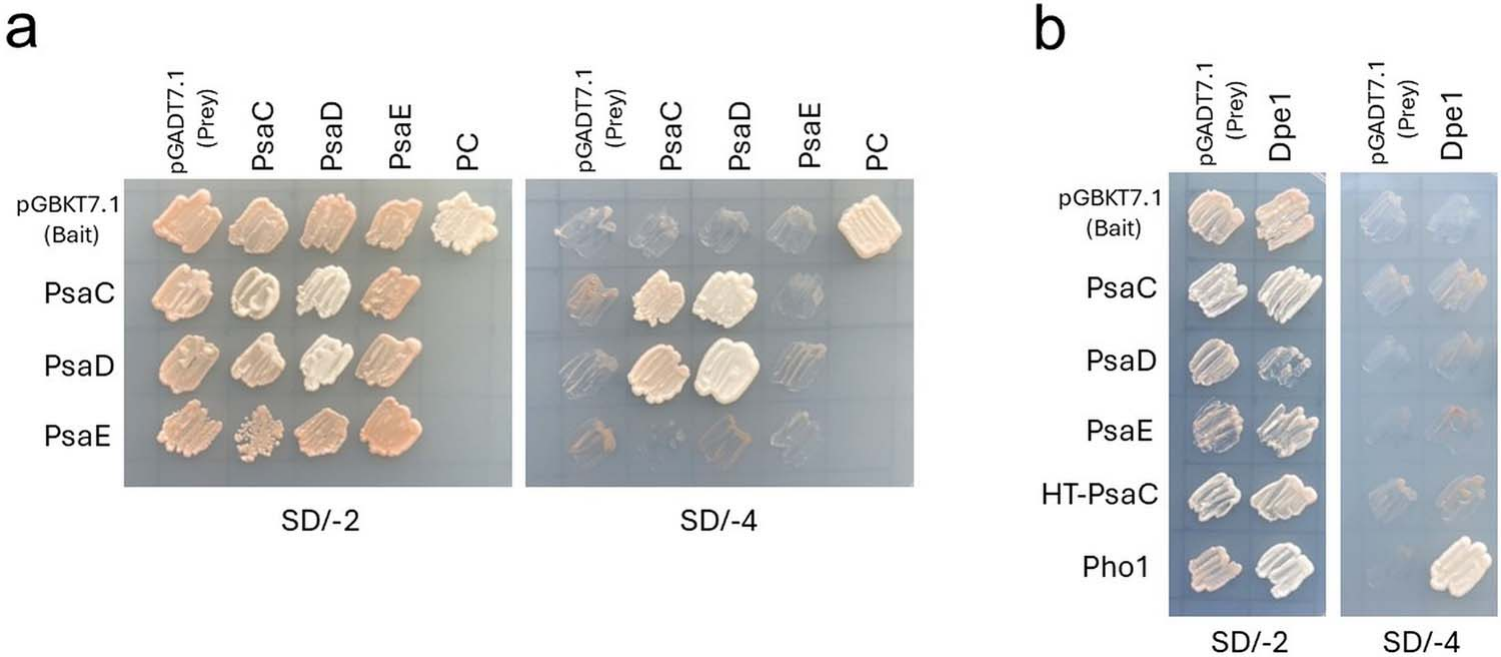
Y2H assay to examine interaction between PSI subunits and Dpe1. Yeast strain AH109 was co-transformed with plasmids encoding bait proteins (PsaC, PsaD, PsaE, and HT-PsaC, Pho1) fused to the GBD and prey proteins (PsaC, PsaD, PsaE, and Dpe1) fused to the GAD. Plasmid names and schematic maps of the expressed proteins are shown in Supplementary Fig. S2. Co-expression of empty vectors pGBKT7.1 or pGADT7.1 served as negative controls, while Pho1 + Dpe1 co-expression was used as a positive control. (a) Protein–protein interactions among PsaC, PsaD, and PsaE were examined. (b) PsaC, PsaD, PsaE, and HT-PsaC were tested for interactions with Dpe1.

**Figure 6:**
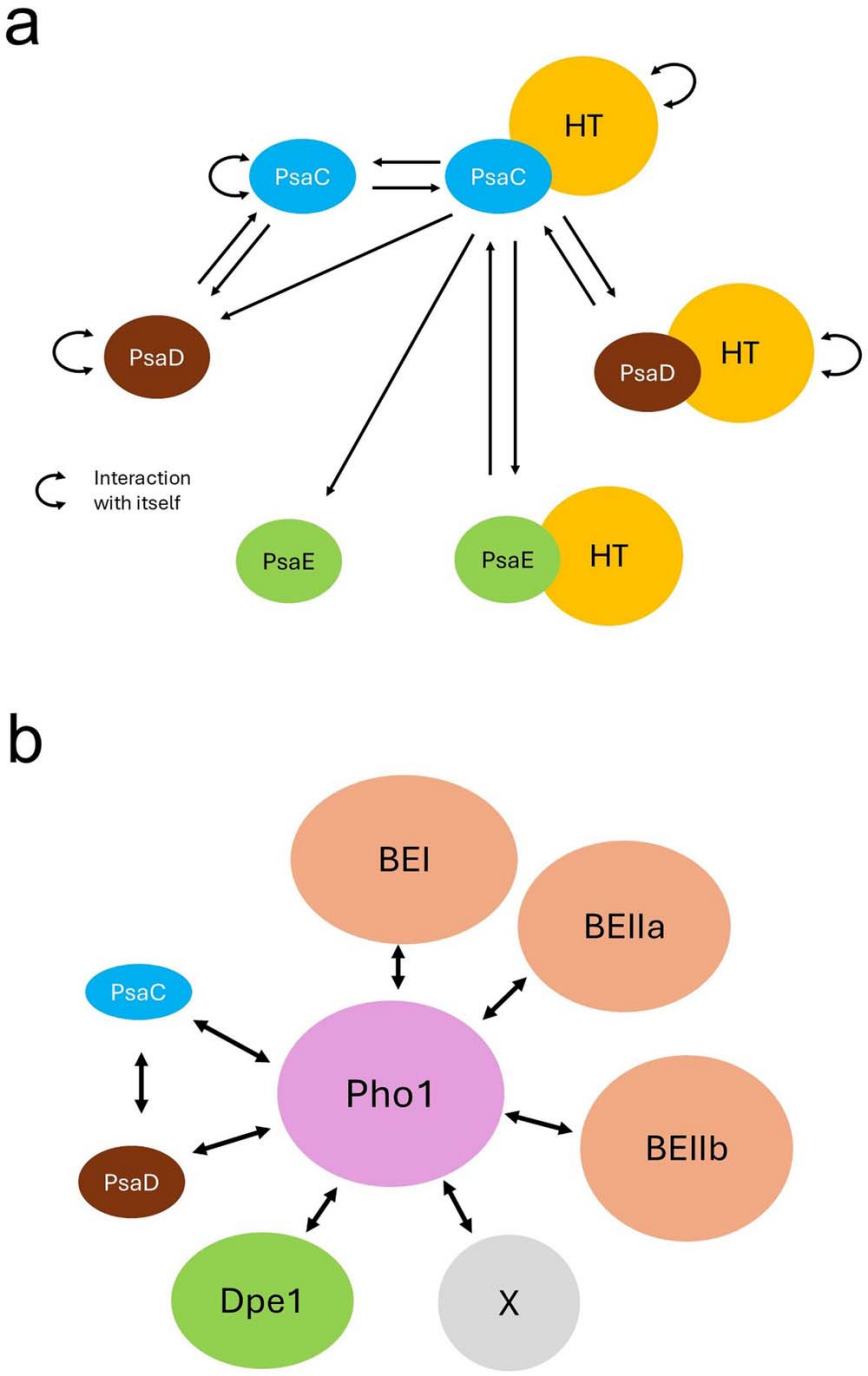
Summary of protein–protein interactions involving Pho1. (a) Schematic representation of the proposed interaction network among stromal PSI subunits. Orange circles indicate HaloTag fusions. Solid lines indicate interactions detected by Y2H assays. Arrows show the direction of combination in the Y2H assay, from GBD fusion (bait) toward GAD fusion (prey). (b) Proposed model of the Pho1 interactome-based on BiFC, pulldown, and Y2H assays, integrated with previously published results. The model illustrates the interactions among Pho1, Dpe1, branching enzymes (BEI, BEIIa, and BEIIb), and the stromal PSI proteins, PsaC and PsaD, along with potential additional partners (X), such as members of the LHC family (Muntaha and Fettke 2025) and, BGC1, the carbohydrate-binding protein essential for formaton of B-type starch granules in developing wheat endosperm (Kamble et al. 2023).

As expected from their spatial arrangement in PSI, PsaC interacted with PsaD (Fig. 5a). However, no interaction was detected between PsaC and PsaE or PsaD and PsaE, which was unexpected considering their close proximity in the PSI structure (Fromme et al. 2003; Naschberger et al. 2024). Y2H analysis further revealed that both PsaC and PsaD self-assembled to form dimers, whereas PsaE did not. None of the PSI subunits, PsaC, PsaD, PsaE, and HaloTag–PsaC, interacted with Dpe1, indicating no direct interaction between PSI and Dpe1 (Fig. 5b). When a HaloTag was fused to the N-termini of these proteins, slight differences in interaction patterns were observed (Supplementary Fig. S4, Fig. 6a). HaloTag–PsaC interacted with all three subunits, indicating that the tag may enhance protein–protein interactions, particularly between PsaC and PsaE. HaloTag–PsaD interacted with HaloTag–PsaC and HaloTag–PsaD, but not with untagged PsaD, PsaE, or HaloTag–PsaE, indicating that the HaloTag fusion interferes with PsaD’s native binding capacity. Neither PsaE nor HaloTag–PsaE showed any self-interaction.

## Discussion

Pho1 plays a significant role in starch metabolism and has been extensively studied for its involvement in starch synthesis and degradation as well as refining starch granule structure (Malinova et al. 2017, Hwang et al. 2020, Li et al. 2024, Seung et al. 2025). A knockdown mutant of rice Pho1 demonstrated that Pho1 is important for starch granule initiation in rice (Satoh et al. 2008). Pho1 is exclusively associated with B-type granule initiation and physically interacts with B-GRANULE CONTENT1 (BGC1), a protein required for both A- and B-type granule initiation in wheat endosperm, suggesting that Pho1 plays an essential role in wheat grain development (Kamble et al. 2023). Gaining insight into Pho1’s protein–protein interactions could significantly advance our understanding of the coordination between starch biosynthesis and its impact on plant growth and development. Previous studies demonstrated that Pho1 interacts with starch branching enzymes (BEI, BEIIa, and/or BEIIb) (Koper et al. 2021a, Nakamura et al. 2012, 2017, Tetlow et al. 2004) and Dpe1 (Hwang et al. 2016b). However, less is known about its potential interaction with other cellular processes, including photosynthesis. Interestingly, our earlier work (Koper et al. 2021) uncovered a potential relationship between starch metabolism and photosynthesis, based on the interaction between Pho1 and the PSI subunit, PsaC, as revealed by Co-IP, pull-down assays, and blue native-PAGE analysis (Koper et al. 2021). We have since confirmed the co-localization of Pho1 and PsaC on thylakoid membranes in rice chloroplasts through double labeling immunocytochemistry at the electron microscope level (Fukuda et al. 2026). These findings indicate that potentially Pho1 physically associates with components of photosynthetic machinery and, thereby, potentially influencing PSI activity or stability. Mutation of the Pho1-encoding *PHS1* in *Arabidopsis* led to defects in chloroplast membrane organization in the *sbe2.1 sbe2.2* background, including reduced grana stacking and a disrupted outer envelope membrane. These results indicate that Pho1 plays a role in maintaining chloroplast structural integrity (Wang et al. 2025). Since the interaction between Pho1 and PsaC appears to be impactful for future studies, more conclusive evidence is warranted. To further explore this interaction, we expressed and purified recombinant Pho1 and PsaC in *Escherichia coli* for in vitro binding assays. However, PsaC proved highly insoluble and aggregated into inclusion bodies. Even with various solubility-enhancing affinity tags such as GST, mCherry, maltose binding protein, and HaloTag, we were unable to obtain intact, soluble PsaC (unpublished data). This suggests that PsaC requires a specific environment—likely provided by its native PSI complex—for structural stability. Our difficulty in purifying PsaC is consistent with its biochemical nature as a [4Fe-4S] cluster-containing protein that is sensitive to oxidative conditions and highly unstable as an apoprotein devoid of the [4Fe-4S] cofactor (Mehari et al. 1995, Antonkine et al. 2002).

BiFC analysis in BY-2 cells revealed that Pho1 interacts with PsaC as well as itself to form its native dimer form (Fig. 1). Because the mature Pho1 sequence (lacking plastid transit peptide) was used and the plastid-coded PsaC naturally lacks a chloroplast transit peptide, the resulting YFP signal was predominantly localized in the cytosol. The fluorescence signal for Pho1–PsaC interaction was weaker compared to the Pho1–Pho1 interaction. Nevertheless, these findings confirm that Pho1 physically interacts with PsaC in living cells.

To further understand the formation of a multiprotein complex, we also investigated whether Pho1 could simultaneously interact with Dpe1, a previously confirmed binding partner (Hwang et al. 2016b, Koper et al., 2021) and PsaC. However, interpretation would be complicated by the use of the Pho1 knockdown mutant BMF136, which still produces low levels of two Pho1 isoforms (Supplementary Fig. S1): a full-length Pho1 and a truncated Pho1 variant missing 53 amino acids at the Pho1 dimer interface (Supplementary Table S1). To eliminate residual Pho1 expression, we generated a *pho1* null line using CRISPR-Cpf1 targeting exon-2. Three independent homozygous T2 lines completely lacking Pho1 protein were confirmed by DNA sequencing and immunoblotting with Pho1-specific antibody (Supplementary Fig. S1). Using these lines, the knockdown mutant (BMF136), and two wild-type varieties (TC65 and Kitaake) as controls, we performed GST-based pulldown assays with protein extracts from whole immature seeds, including pericarp and endosperm tissues, to obtain higher levels of PsaC than when using endosperm alone. When immobilized GST-GFP (negative control) was incubated with soluble seed proteins and eluted, neither Dpe1 nor PsaC was detected in the eluates (Fig. 2b). GST-Dpe1 also pulled down Pho1 and PsaC, but only in wild-type samples, not in BMF136 or Pho1 null mutants (Fig. 2c). In contrast, when GST-Pho1 was used as bait, both Dpe1 and PsaC were detected in the eluates of all plants (Fig. 2d). These results demonstrate that Pho1 can independently bind both Dpe1 and PsaC, and is essential for the potential formation of a Pho1–Dpe1–PsaC ternary complex.

It was surprising that GST-Dpe1 failed to pull down any Pho1 proteins from BMF136, although this knockdown line still expresses very low levels of both full-length and truncated Pho1 variants. As Dpe1 and PsaC do not directly interact with each other (Fig. 5b), Pho1 may serve as a bridging protein for complex formation. We should point out that Pho1’s interaction with PsaC was only observed when freshly prepared protein extracts were used and a highly sensitive detection kit was used. When the samples were stored after extraction and used later, no PsaC signal was detected (data not shown). This indicates that PsaC is highly unstable in its free form in the extraction buffer, even when protease inhibitors and reducing agent are present. This instability is consistent with our observations from bacterial expression of recombinant HaloTag-PsaC and GST-PsaC, where the PsaC region in the soluble fusion proteins underwent degradation during expression (Supplementary Fig. S5).

Further confirmation demonstrating a specific interaction between Pho1 and PsaC was provided by Y2H assays (Fig. 4). The truncated variant Pho1∆L80, lacking the L80 domain, also interacted with PsaC, suggesting the L80 domain is not essential for this binding. The strength of interaction of PsaC with the two Pho1 proteins varied considerably depending on the presence of a HaloTag on PsaC. In the absence of the HaloTag, PsaC exhibited a stronger interaction with Pho1ΔL80 than with Pho1. Such a stronger association between PsaC with Pho1ΔL80 than Pho1 was also evident in PsaC pulldown studies published earlier (Koper et al. 2021). However, this difference was not observed when PsaC was fused to the HaloTag, as HaloTag–PsaC interacted about equally with both Pho1 and Pho1ΔL80. Interestingly, a distinct interaction pattern was seen for PsaC with the catalytic-null mutants, Pho1[K823A] and Pho1∆L80[K823A]. Both null enzymes showed no significant interaction with PsaC, indicating that the K823A mutation impairs Pho1’s ability to bind PsaC. This reduction in binding efficiency was reversed when PsaC was fused at its N-terminus to HaloTag. One possible basis for the enhanced interaction of the HaloTag-PsaC with the two Pho1 variant proteins is increased stability of HaloTag-PsaC compared to PsaC. However, this is unlikely, as the HaloTag-PsaC was readily degraded after expression in *E. coli*  Supplementary Fig. S5. A more likely explanation is that the presence of a HaloTag moiety stabilizes a structural conformation in PsaC that enhances its affinity for Pho1 as well as the two catalytic null Pho1 proteins, potentially compensating for the structural disturbance caused by the mutation. The molecular basis of these contrasting effects remains unclear and warrants further investigation through structural and biochemical analyses. The Y2H assay in this study further revealed that Pho1 interacts with PsaD, but not with PsaE, suggesting that Pho1 may also form a protein complex with PsaD. Unlike PsaC, the HaloTag–PsaD did not interact with Pho1 or Pho1ΔL80, indicating that the HaloTag interfered with assembly of PsaD with Pho1.

The ability of HaloTag–PsaC to interact with the catalytic-null mutants, Pho1[K823A] and Pho1∆L80[K823A], may afford us the exceptional opportunity to study specific aspects of Pho1 biology. As aforementioned, rice lines expressing Pho1ΔL80 exhibit enhanced plant growth, early flowering, and increased biomass, grain size, and grain yields (Koper et al. 2021). These enhanced agronomic traits are a product of Pho1ΔL80-mediated enhancement of starch metabolism and photosynthesis, the latter due to PSI modulation (Koper et al. 2021). If Pho1∆L80[K823A] is able to interact with PSI-associated PsaC, we suspect that it will stimulate PSI activity similar to that generated by Pho1∆L80. Indeed, young BMF136 rice lines expressing Pho1∆L80[K823A] display early seedling growth similar to that seen for Pho1∆L80 plants (unpublished data). If true, then plant growth properties can be studied solely by the impact of Pho1∆L80[K823A] on PSI in the absence of the Pho1-mediated starch metabolism.

While Pho1 and Pho1∆L80 interacted with PsaC, the absence of the L80 region in Pho1 markedly affected photosynthetic properties. Expression of Pho1∆L80 in the BMF136 mutant enhanced plant growth and altered photosynthetic efficiency compared to wildtype and BMF136, and Pho1 transgenic plants, indicating that the L80 region contributes to the regulation of PSI activity (Koper et al. 2021). However, Y2H assays using the isolated L80 peptide and its adjacent residues showed no interaction with the stromal subunits of PSI: PsaC, HaloTag-PsaC, PsaD, HaloTag-PsaD, PsaE, or HaloTag-PsaE (Supplementary Fig. S5), suggesting that this domain influences Pho1 function through an indirect regulatory mechanism rather than by binding to PSI components.

Consistent with our previous findings (Hwang et al. 2016b), Y2H analysis confirmed that Dpe1 interacts with Pho1 (Fig. 3a). It also interacts with Pho1∆L80 and their corresponding catalytic mutant forms. The absence of interaction between Dpe1 and the isolated L80 domain, with or without its adjacent region (Fig. 3b) further supports that the L80 domain is not required for the Pho1–Dpe1 interaction. These results also indicate that catalytic inactivation has no impact on complex formation between Pho1 and Dpe1.

As expected, PsaC interacted with PsaD (Fig. 5), consistent with their proximity within the PSI complex (Fromme et al. 2003, Jensen et al. 2007). However, the absence of its interaction between PsaC and PsaE in this assay was unanticipated, given that all three subunits are structurally intertwined on the stromal side of PSI (Jensen et al. 2007). Interestingly, both PsaC and PsaD displayed self-assembly as a dimer in the Y2H system (Figs. 5 and 6a), suggesting their potential to form homo-oligomers. In contrast, PsaE did not show any self-interaction, consistent with its proposed peripheral positioning and transient role in the PSI complex.

The addition of an N-terminal HaloTag to these PSI subunits slightly altered their interaction profiles (Supplementary Fig. S4). Unlike the native form, HaloTag–PsaC was now able to interact with PsaE as well as with PsaD, indicating that the HaloTag may enhance or stabilize subunit interactions, particularly between PsaC and PsaE. Conversely, HaloTag–PsaD interacted only with HaloTag–PsaC and HaloTag–PsaD, but not with the untagged forms of PsaD and PsaE or HaloTag–PsaE, suggesting that the HaloTag interferes with native PsaD interactions, possibly through steric hindrance or altered protein folding. Our findings suggest that PsaC plays a central role in mediating the assembly of the PsaC–PsaD–PsaE heterocomplex. Taken together, these results indicate that PsaC and PsaD can form multimers and likely cooperate to recruit PsaE to form a stable PsaC–PsaD–PsaE stromal subcomplex. PsaC and PsaD appear to function as anchoring subunits that bridge Pho1 to the PSI complex, supporting proper assembly and enabling functional connection between Pho1 and the electron transfer machinery. It remains unclear whether Pho1 interacts with monomeric or oligomeric forms of PsaC and PsaD. However, their ability to self-associate may influence its interaction properties prior to or during PSI assembly. Further biochemical and structural studies are required to determine the stoichiometry and conformational states of PsaC and PsaD complexes and to clarify how Pho1 engages with these different oligomeric forms. Taken together, our findings support a model in which Pho1 assembles into a dynamic protein complex with PsaC, PsaD, and Dpe1, potentially acting at the interface between photosynthesis and starch metabolism and serving a central role in mediating these interactions (Fig. 6b). This complex may also include additional key components, such as branching enzymes (Tetlow et al. 2004, Nakamura et al. 2012) and additional photosynthesis proteins (X in Fig. 6b) as suggested by the Y2H study by Muntaha and Fettke (2025).

Our findings demonstrate that Pho1 physically interacts with PsaC, PsaD, and Dpe1. The complex potentially interacts with other proteins such as branching enzymes and light-harvesting complex (LHC) family (Muntaha and Fettke 2025), forming a multi-protein complex that may function at the intersection of photosynthesis and starch metabolism. The interaction between Pho1 and PsaC is strongly supported by multiple complementary approaches in this study—including pulldown assays, BiFC, and Y2H—as well as by our previously published data, thereby reinforcing the biological relevance of this association. Despite these advances, the precise mechanism by which Pho1 modulates PSI function remains unresolved, particularly considering the substantial size disparity between Pho1 and individual stromal PSI subunits (Fromme et al. 2003, Hwang et al. 2010, Jensen et al. 2007). The dynamic coordination between photosynthesis and starch biosynthesis could be essential for maintaining energy balance, promoting growth, and potentially enhancing stress tolerance in plants. Future research should prioritize mapping the specific interaction domains between Pho1 and PSI subunits, particularly PsaC and PsaD, using approaches such as mutagenesis, domain truncation, and structural modeling. It will also be crucial to elucidate the regulatory role of the L80 domain, which appears to modulate Pho1’s function without directly binding to PSI subunits. Additionally, determining whether Pho1 interacts with free stromal PsaC and PsaD, and understanding how these interactions impact PSI assembly and electron transfer efficiency will be key to elucidating Pho1’s role in photosynthetic regulation. It will be also interesting to examine whether the Pho1–Dpe1–PsaC–PsaD multimeric complex exists and interacts with additional photosynthesis proteins such as members of the LHC family (Muntaha and Fettke 2025). Ultimately, a deeper understanding of how Pho1 coordinates metabolic and photosynthetic pathways could reveal novel strategies to enhance plant productivity and energy use efficiency—important goals for both basic research and agricultural innovation.

## Materials and Methods

### DNA manipulation of rice PsaC, PsaD, PsaE, Dpe1, and Pho1 variants

Total DNA was extracted from 4-week-old rice seedlings (Hwang and Kim 2000). Oligonucleotides used in this study are listed in Supplementary Table S2, and an overview of plasmid DNA construction is provided in Supplementary Table S3. The coding sequences of PsaC, PsaD, and PsaE were amplified by PCR using Pfu DNA polymerase (Thermo Fisher). The gene-specific primers (Supplementary Table S2) were designed to include restriction enzyme sites for *Nco*I and *Xho*I at the 5′ and 3′ ends, respectively. Aside from PsaC and PsaD, PsaE contains an intron sequence that was removed from the original clone to express the mature form of the protein. pGADT7 and pGBKT7 (Clontech’s Matchmaker™ Systems) were modified by removing an upstream *Nco*I site to produce pGADT7.1 and pGBKT7.1, respectively. These plasmid vectors carry a single *Nco*I site at the 5′ end and multiple other unique sites, including *Kpn*I and *Sac*I at the 3′ ends. DNA sequences of Pho1 and Pho1∆L80 (Hwang et al. 2016a, 2016b) were subjected to site-directed mutagenesis to substitute lysine at position 823, a PLP binding site (Shoaib et al. 2021), with alanine, generating the catalytic-null Pho1[K823A] and Pho1∆L80[K823A], respectively. These catalytic mutants exhibited no detectable enzyme activity when recombinant proteins, expressed and purified, were assayed using Glc1-P and amylopectin as substrates as previously described (Hwang et al. 2010, 2016a) Supplementary Fig. S6. Sequences coding for Pho1 catalytic mutants, along with wild-type Pho1 and Pho1∆L80, were digested with *Nco*I and *Kpn*I and individually cloned into pGADT7.1. The L80 region and an extended L80 region containing surrounding sequence (L80E) (Supplementary Fig. S2) were amplified by PCR and cloned into pGADT7.1 and pGBKT7.1 with or without a HaloTag at their N-terminal ends. The coding sequences of PsaC, PsaD, PsaE, and Dpe1 (Hwang et al. 2016b) were digested with *Nco*I and *Sac*I and ligated into the corresponding sites of pGBKT7.1. The ligation mixtures were transformed into chemically competent *E. coli* XL10-Gold cells, and transformants were selected on LB plates containing appropriate antibiotics. Positive colonies were screened by restriction enzyme digestion and confirmed by Sanger sequencing. Schematic maps of the expression constructs are presented in Supplementary Fig. S2.

### Bimolecular fluorescence complementation assays

BiFC assays were conducted to investigate the interaction between Pho1 and PsaC in live cells. The full-length mature sequences of Pho1 and PsaC were cloned into both pSAT1-nEYFP-C1 and pSAT1-cEYFP-C1-B (Arabidopsis Biological Resources Center) to fuse them with the N-terminal and C-terminal fragments of the EYFP fluorescent protein. Pairs of the resulting constructs (30–40 *μ*g each), pSH1135 (nEYFP-Pho1), pSH1155 (cEYFP-Pho1), and pSH1141 (cEYFP-PsaC) together with their respective empty vectors (Supplementary Fig. S2, Supplementary Table S3), were co-transfected into 300 *μ*L of BY-2 protoplast using an equal volume of PEG solution [40% PEG 4000, 0.4 M mannitol, 0.1 M Ca(NO_3_)_2_], following the method described in a previous study (Tian et al. 2018). Cells were incubated at room temperature for 20 min, and PEG solution was removed by washing with W5 solution (2 mM MES–NaOH, pH 5.7, 150 mM NaCl, 125 mM CaCl_2_, 5 mM KCl, 0.4 M mannitol). The transfected protoplasts were then incubated at 26°C for 16 h in protoplast culture medium before observation. Fluorescence reconstitution, indicating protein–protein interaction, and light images were visualized using a Zeiss LSM 510 Meta confocal microscope. Controls included co-transfection of each protein construct with an empty vector to assess background fluorescence.

### Protein expression and purification of glutathione S-transferase-tagged proteins

The coding sequences of Dpe1 and Pho1 were cloned into pSH1440 (GST-GFP), which was derived from pGEX-2T (GE Healthcare), generating pSH1441 and pSH1442, respectively (Supplementary Fig. S2, Supplementary Table S3). *Escherichia coli* EA3457 cells (Hwang et al. 2016a) transformed with the plasmids were grown in NZCYM medium supplemented with 200 mg/L penicillin G and 34 mg/L chloramphenicol at 37°C. When the optical density at 600 nm reached 0.6–0.8, protein expression was induced with 0.1 mM isopropyl β-D-1-thiogalactopyranoside at 28°C for 16 h. After induction, cells were harvested by centrifugation at 4000× g for 30 min, resuspended in lysis buffer (25 mM Tris–HCl, 150 mM NaCl, 1 mM EDTA, 2 mM NaF, 1× proteinase inhibitors, 1 mM PMSF, pH 7.5) containing 200 ug/mL lysozyme, and lysed by sonication (Sonic Dismembrator, Fisher Scientific) after 1 h incubation on ice. The lysate was clarified by centrifugation at 15 000 × g for 10 min at 4°C. The supernatant was applied to a glutathione-agarose (Invitrogen) pre-equilibrated with binding buffer (25 mM Tris–HCl, 150 mM NaCl, 1 mM EDTA, 2 mM DTT, pH 7.5). After binding, the column was washed with 8 bed volumes of binding buffer to remove non-specifically bound proteins. Protein was eluted with elution buffer (binding buffer +5 mM glutathione). Eluted fractions were analyzed by SDS-polyacrylamide gel electrophoresis (Laemmli 1970), and GST-fusion proteins were precipitated in 70% ammonium sulfate (pH 7). Protein pellets were resuspended in storage buffer consisting of 50 mM Tris–HCl, pH 7.5, 150 mM NaCl, 1 mM Tris(2-carboxyethyl)phosphine (TCEP), 10% glycerol. Protein concentration was determined using the Bradford assay (Bradford 1976) using BSA as standard.

### CRISPR-based production of phosphorylase null mutants in rice

To generate Pho1 knockout rice lines completely lacking Pho1 protein expression, we employed CRISPR-Cpf1 genome editing. A single-guide RNA (sgRNA) was designed to specifically target exon-2 of the Pho1 coding region. For genome editing, a binary CRISPR-Cpf1 expression vector pSH916 was constructed by combining elements from pYPQ230 (Tang et al. 2017) and pRGEB31 (Xie and Yang 2013). An oligonucleotide mixture containing 50 pmol each of Pho1-cR1-F (5′-AGATACCTCTGAAGGCTTACCATGCAA-3′) and Pho1-cR1-R (5′-TGGAGACTTCCGAATGGTACGTTAAAA-3′) was boiled for 10 min and then allowed to anneal by gradual cooling to room temperature. The resulting double-stranded DNA fragment was ligated into *Bsa*I-digested pSH924A (Supplementary Fig. S7a). The sgRNA expression cassette from the resulting plasmid pSH984T was amplified by PCR using U6B2-BsaF (5′-AAGGTCTCTGGACCGATCTGCCGCCGGATCATG-3′) and U6B2t-BsaR (5′-AGAGGTCTCAAAACTGACCATTTGTGGGTCTGT-3′) with *Pfu* DNA polymerase, and then cloned into the *Bsa*I sites of pSH916, placing it under the control of the maize ubiquitin (Ubi) promoter. The final plasmid construct, pSH984 (Supplementary Fig. S7b), was introduced into *Agrobacterium tumefaciens* strain AGL1, which was then used to infect embryogenic calli derived from rice (*Oryza sativa* cv. Kitaake). Transformation, selection, and regeneration of transgenic plants were carried out according to the protocols essentially described in earlier studies (Toki 1997, Hamada et al. 2003).

### Pulldown assay

Developing seeds were harvested at 7 days after flowering (DAF) from wild-types (cv. Kitaake and cv. TC65), BMF136 (Satoh et al. 2008) and transgenic rice plants, and immediately stored at −80°C until use. Whole seeds were ground to a fine powder in liquid nitrogen using a pre-chilled mortar and pestle, whereupon 0.5 g of the powdered tissue was transferred into a pre-cooled 2-mL microcentrifuge tube and flash-frozen again in liquid nitrogen. To ensure thorough pulverization, we used a bead mill homogenizer (Omni International) with two cycles of 30 s at 4 m/sec. The resulting powder was resuspended in 0.5 mL of ice-cold lysis buffer consisting of 50 mM Tris–HCl (pH 7.5), 150 mM NaCl, 1 mM EDTA, 1 mM NaF, 0.1 mM TCEP, 0.05% (v/v) Nonidet P-40, 0.05% (v/v) Triton X-100, 1 mM PMSF, and 1× proteinase inhibitor cocktail. The mixture was vortexed thoroughly to solubilize proteins, centrifuged at 300× g for 5 min at 4°C and the supernatant was carefully collected. This extraction step was repeated with an additional 0.5 mL of binding buffer. The samples were centrifuged again at 15 000× g for 10 min at 4°C, and the final supernatant was collected.

For GST pull-down assays, 100 *μ*g each of the following GST-tagged fusion proteins, GST-GFP (control), GST-Pho1, and GST-Dpe1 (Supplementary Fig. S2, Supplementary Table S3), was pre-bound to 25 *μ*L glutathione agarose resin (Invitrogen Molecular Probes) in excess volume of binding buffer consisting of 50 mM Tris–HCl (pH 7.5), 150 mM NaCl, 1 mM EDTA, 1 mM NaF, 0.1 mM TCEP, 1 mM PMSF, 1× proteinase inhibitor cocktail and incubated at 4°C for 30 min with gentle agitation. The resin was then pelleted by centrifugation at 100× g for 10 s, and the supernatant was discarded. This washing was repeated four times using binding buffer to remove any unbound or loosely associated proteins. After washing, 100 *μ*L of protein extract was added to the resin, followed by incubation at 4°C for 2 h to allow specific interaction. After binding, the resin was transferred to small gravity flow columns and washed four times with ice-cold binding buffer to remove nonspecific interactions. Bound proteins were eluted using an elution buffer composed of the binding buffer supplemented with 5 mM reduced glutathione and 2 M urea to dissociate protein complexes from the resin. Eluted samples were collected, and 15 uL of each eluate was loaded onto a 12.5% SDS-PAGE gel and resolved using a Tris-glycine running buffer system (Laemmli 1970) for subsequent detection of Pho1 and Dpe1. For PsaC detection, eluates were separated on 12.5% SDS-PAGE gels using the Tris-tricine buffer system (Schägger 2006). Proteins in the gels were transferred to nitrocellulose (Pho1 and Dpe1 detection) or to Immobilon-PSQ (PsaC detection). Immunoblotting was performed with polyclonal anti-rabbit antibodies raised against denatured, full-length recombinant Pho1 and Dpe1 proteins (Hwang et al. 2016b, Koper et al. 2021) in 5% nonfat dry milk prepared in TBST (20 mM Tris–HCl, pH 7.5, 150 mM NaCl, 0.05% Tween-20). For PsaC detection, a commercially available antibody (PhytoAB, Inc.) was used at a 1:500 dilution. Signals were visualized using SuperSignal West Pico PLUS Chemiluminescent Substrate (Thermo Scientific) for Pho1 and Dpe1, and SuperSignal West Femto Chemiluminescent Substrate for PsaC, and images were captured with the Amersham Imager 600 system.

### Yeast two-hybrid assays

Y2H assays were performed to investigate the interactions among Pho1 variants and potential partner proteins. The *Saccharomyces cerevisiae* strain AH109 was used as the host for transformation. Preparation of electrocompetent cells and electroporation were performed as described previously (Suga and Hatakeyama 2003) with minor modifications. Yeast cells were cultured in YPDA medium (2% bacto-peptone, 1% yeast extract, 2% glucose, 40 mg/L D-adenine hemisulfate) to the late logarithmic phase, harvested by centrifugation at 4000 rpm for 5 min at 4°C, and then washed three times with ice-cold sterile water under the same centrifugation conditions. To prepare cells for transformation, they were then washed once with freeze–thaw buffer (10 mM HEPES-NaOH, pH 7.5, 1 M D-sorbitol, 10 mM CaCl_2_), then resuspended in 1/20th of the original culture volume using the same buffer. The cell suspension was aliquoted and stored at −80°C until use. Immediately before transformation, aliquots were thawed at 37°C for 5 min and then placed on ice. Cells were washed twice with 1 mL of 1 M D-sorbitol and resuspended in 0.2 mL of the same buffer. For each transformation, 0.5–1 *μ*g of each plasmid (e.g. pGBKT7 and pGADT7 vectors containing the respective coding sequences) was mixed with the cell suspension and transferred to a 2-mm electroporation cuvette. Electroporation was carried out using a Bio-Rad MicroPulser Electroporator with a single pulse at 2.5 kV. Immediately after pulsing, 1 mL of a 1:1 mixture of 1 M D-sorbitol and YPDA medium was added to the cuvette. The mixture was transferred to a 1.5-mL microcentrifuge tube and incubated at 30°C for 1 h with shaking. Following incubation, the cells were centrifuged at 4000 rpm for 1 min, washed once with 1 M D-sorbitol, and resuspended in 100 *μ*L of 1 M D-sorbitol. Co-transformed yeast cells were plated on SD/−Leu/−Trp (SD/−2) selective medium to confirm the presence of both plasmids. Plates were incubated at 30°C for 3–5 days until colonies formed. To assess protein–protein interactions, the transformants were further cultured on SD/−Leu/−Trp/−His/−Ade (SD/−4) plates. A positive interaction was indicated by robust colony growth on SD/−4 medium, reflecting activation of reporter genes driven by physical interaction between the proteins fused to the GAL4 DNA-binding and activation domains. In contrast, no growth was observed in control experiments where yeast cells were transformed with the target protein and either pGBKT7.1 or pGADT7.1 alone, to rule out auto-activation.

## Supplementary Material

Supplementary_material_pcag063

## Data Availability

All data supporting the findings of this study are available within the article and its Supplementary Data file.
