# Supplementary material for: Starch phosphorylase acts as a pivotal scaffold connecting starch metabolism enzymes with photosystem I subunits
Source: Plant Cell Physiol. 2026 May 14;67(8):1430–41. doi: 10.1093/pcp/pcag063 (PMC13545646; doi:10.1093/pcp/pcag063)
Supplement: Supplementary file 1 [file supplementary_material_pcag063.zip › Supplementary Fig. S1.docx]

a.

b.

**Supplementary Figure S1. DNA sequence analysis (a) and immunodetection of Pho1 (b) in CRISPR-Cpf1 transgenic rice plants.** (a) DNA sequences of *PHO1* exon2 in wildtype (cv. Kitaake) and T1 transgenic lines were analyzed. Lines #24, #72, and #92 contain nucleotide deletions of 28 bp, 8 bp, and 19 bp, respectively, in exon 2 of the rice Pho1 gene. These deletions result in truncated Pho1 proteins consisting of only 107, 107, and 110 amino acid residues, respectively (the wildtype Pho1 contains 978 amino acids before maturation). (b) Total proteins were extracted from four individual dry seeds (T2 generation) from each T1 transgenic line. Each seed was ground into a fine powder, and proteins were solubilized in 200 µL of urea-SDS sample buffer. Ten microliters of each extract were resolved by 10% SDS-PAGE and electroblotted onto an Immobilon-PS^Q^ membrane (Millipore). The membrane was blocked for 1 h at room temperature with 5% (w/v) non-fat dry milk in TBST buffer (20 mM Tris-HCl, pH 7.5, 150 mM NaCl, 0.05% Tween-20). Pho1 protein was detected using a Pho1-specific primary antibody (1:1000) by incubating overnight at 4˚C with shanking. After washing with TBST, the membrane was incubated with an HRP-conjugated anti-goat secondary antibody (1:5000) for 1 h at room temperature. Signal was visualized using SuperSignal West Pico PLUS Chemiluminescent Substrate (Thermo Scientific) and imaged with the Amersham Imager 600 system. Samples analyzed were dry seeds from wildtype cv. Kitaake (WT), BMF136, and two T1 transgenic plants each from independent lines #24, #72, and #92.
