# Supplementary material for: Starch phosphorylase acts as a pivotal scaffold connecting starch metabolism enzymes with photosystem I subunits
Source: Plant Cell Physiol. 2026 May 14;67(8):1430–41. doi: 10.1093/pcp/pcag063 (PMC13545646; doi:10.1093/pcp/pcag063)
Supplement: Supplementary file 1 [file supplementary_material_pcag063.zip › Supplementary Fig. S2.docx]

**PsaC**

MSHSVKIYDTCIGCTQCVRACPTDVLEMIPWDGCKAKQIASAPRTEDCVGCKRCESACPTDFLSVRVYLGPETTRSMALSY

**PsaD (mature form)**

MAAATEEKKPAPAGFVPPQLDPNTPSPIFGGSTGGLLRKAQVEEFYVITWTSPKEQVFEMPTGGAAIMREGPNLLKLARKEQCLALGTRLRSKYKINYQFYRVFPNGEVQYLHPKDGVYPEKVNAGRQGVGQNFRSIGKNVSPIEVKFTGKNVFDI

**PsaE (mature form)**

MAEEEAAAPPPPPPPAAEEKPAEAEAAVATKEPAAAKPPPIGPKRGTKVKILRRESYWYNGTGSVVTVDQDPNTRYPVVVRFAKVNYAGVSTNNYALDEIQEVK

**L80**

IDLPDSIAKLFVKPKEKKESPAKLKEKLLVKSLEPSVVVEEKTVSKVEINEDSEEVEVDSEEVVEAENEDSEDELDPFVK

**L80E (L80 is underlined)**

LMNIIISKYGTEDTSLLKKKIKEMRILDNIDLPDSIAKLFVKPKEKKESPAKLKEKLLVKSLEPSVVVEEKTVSKVEINEDSEEVEVDSEEVVEAENEDSEDELDPFVKSDPKLP

**Figure S2. Schematic representation of plasmid vectors used in this study for bacterial protein expression, BiFC, pulldown, and Y2H assays.** Asterisk denotes the K823A substitution. H, 6xhistidine tag. Protein sequences of PsaC, PsaD (mature form), PsaE (mature form), L80, and L80E are also shown.
