# Supplementary material for: Starch phosphorylase acts as a pivotal scaffold connecting starch metabolism enzymes with photosystem I subunits
Source: Plant Cell Physiol. 2026 May 14;67(8):1430–41. doi: 10.1093/pcp/pcag063 (PMC13545646; doi:10.1093/pcp/pcag063)
Supplement: Supplementary file 1 [file supplementary_material_pcag063.zip › Supplementary Fig. S6.docx]

|  | Relative Activity (%) |
| --- | --- |
| Pho1 | 100 |
| Pho1∆L80 | 80.6364 |
| Pho1[K823A] | 0.05 |
| Pho1∆L80[K823A] | 0.05 |

**Supplementary Figure S6. Enzyme activities of Pho1 and its variant proteins**. Recombinant Pho1, Pho1Δ80, Pho1[K823A], and Pho1Δ80[K823A] proteins were expressed in *E. coli* and purified using affinity chromatography as previously described (Hwang et al. 2010, 2016b). Starch phosphorylase activities were assayed at room temperature in the starch synthesis direction. Reaction mixtures contained 0.2% (w/v) amylopectin and 20 mM glucose 1-phosphate in assay buffer, and reactions were initiated by enzyme addition. Product formation was quantified spectrophotometrically using the PAMb method. Specific activities for each variant were normalized to that of wild-type Pho1 and are presented as relative avtivities. Data represent the mean (± SD) of three independent measurements.
