# Supplementary material for: Starch phosphorylase acts as a pivotal scaffold connecting starch metabolism enzymes with photosystem I subunits
Source: Plant Cell Physiol. 2026 May 14;67(8):1430–41. doi: 10.1093/pcp/pcag063 (PMC13545646; doi:10.1093/pcp/pcag063)
Supplement: Supplementary file 1 [file supplementary_material_pcag063.zip › Supplementary Fig. S7.docx]

a.

AAGCTTTAGATGTCGATCTGCCGCCGGATCATGAACCAACGGCCTGGCTGTATTTGGTGGTTGTGTAGGGAGATG

HindIII

GGGAGAAGAAAAGCCCGATTCTCTTCGCTGTGATGGGCTGGATGCATGCGGGGGAGCGGGAGGCCCAAGTACGTGCACGGTGAGCGGCCCACAGGGCGAGTGTGAGCGCGAGAGGCGGGAGGAACAGTTTAGTACCACATTGCCCAGCTAACTCGAACGCGACCAACTTATAAACCCGCGCGCTGTCGCTTGTGTTGAATTTCTACTAAGTGTAGATAGAGAC

*Bsa*I

CATGGTCTCATTTTTTTTTGAGATTTCCATCCAGGTCCCTGAGTTACAAGTATTTTCGTTGCAGATATGCCCCTCCGC

*Bsa*I

CTATTACGAATCTTACAATTCGGTCCTTTTTCCACGTTTTCTTTTCAGTTGCACCCTCAATCTCTCCCTTTGCAGACAGACCCACAAATGGTCAATTTTGTGAATTC

*Eco*RI

b.

CGATCTGCCGCCGGATCATGAACCAACGGCCTGGCTGTATTTGGTGGTTGTGTAGGGAGATGGGGAGAAGAAAAGCCCGATTCTCTTCGCTGTGATGGGCTGGATGCATGCGGGGGAGCGGGAGGCCCAAGTACGTGCACGGTGAGCGGCCCACAGGGCGAGTGTGAGCGCGAGAGGCGGGAGGAACAGTTTAGTACCACATTGCCCAGCTAACTCGAACGCGACCAACTTATAAACCCGCGCGCTGTCGCTTGTGTTGAATTTCTACTAAGTGTAGATACCTCTGAAGGCTTACCATGCAATTTTTTTTTGAGATTTCCATCCAGGTCCCTGAGTTACAAGTATTTTCGTTGCAGATATGCCCCTCCGCCTATTACGAATCTTACAATTCGGTCCTTTTTCCACGTTTTCTTTTCAGTTGCACCCTCAATCTCTCCCTTTGCAGACAGACCCACAAATGGTCAGTTTTTAGCGCGTGCATGCCAAACTGAAGGCGGGAAACGACAATCTGATCCAAGCTCAAGCTGCTCTAGCATTCGCCATTCAGGCTGCGCAACTGTTGGGAAGGGCGATCGGTGCGGGCCTCTTCGCTATTACGCCAGCTGGCGAAAGGGGGATGTGCTGCAAGGCGATTAAGTTGGGTAACGCCAGGGTTTTCCCAGTCACGACGTTGTAAAACGACGGCCAGTGCCAAAGCTTGCATGCCTGCAGTGCAGCGTGACCCGGTCGTGCCCCTCTCTAGAGATAATGAGCATTGCATGTCTAAGTTATAAAAAATTACCACATATTTTTTTTGTCACACTTGTTTGAAGTGCAGTTTATCTATCTTTATACATATATTTAAACTTTACTCTACGAATAATATAATCTATAGTACTACAATAATATCAGTGTTTTAGAGAATCATATAAATGAACAGTTAGACATGGTCTAAAGGACAATTGAGTATTTTGACAACAGGACTCTACAGTTTTATCTTTTTAGTGTGCATGTGTTCTCCTTTTTTTTTGCAAATAGCTTCACCTATATAATACTTCATCCATTTTATTAGTACATCCATTTAGGGTTTAGGGTTAATGGTTTTTATAGACTAATTTTTTTAGTACATCTATTTTATTCTATTTTAGCCTCTAAATTAAGAAAACTAAAACTCTATTTTAGTTTTTTTATTTAATAATTTAGATATAAAATAGAATAAAATAAAGTGACTAAAAATTAAACAAATACCCTTTAAGAAATTAAAAAAACTAAGGAAACATTTTTCTTGTTTCGAGTAGATAATGCCAGCCTGTTAAACGCCGTCGACGAGTCTAACGGACACCAACCAGCGAACCAGCAGCGTCGCGTCGGGCCAAGCGAAGCAGACGGCACGGCATCTCTGTCGCTGCCTCTGGACCCCTCTCGAGAGTTCCGCTCCACCGTTGGACTTGCTCCGCTGTCGGCATCCAGAAATTGCGTGGCGGAGCGGCAGACGTGAGCCGGCACGGCAGGCGGCCTCCTCCTCCTCTCACGGCACCGGCAGCTACGGGGGATTCCTTTCCCACCGCTCCTTCGCTTTCCCTTCCTCGCCCGCCGTAATAAATAGACACCCCCTCCACACCCTCTTTCCCCAACCTCGTGTTGTTCGGAGCGCACACACACACAACCAGATCTCCCCCAAATCCACCCGTCGGCACCTCCGCTTCAAGGTACGCCGCTCGTCCTCCCCCCCCCCCCCCTCTCTACCTTCTCTAGATCGGCGTTCCGGTCCATGGTTAGGGCCCGGTAGTTCTACTTCTGTTCATGTTTGTGTTAGATCCGTGTTTGTGTTAGATCCGTGCTGCTAGCGTTCGTACACGGATGCGACCTGTACGTCAGACACGTTCTGATTGCTAACTTGCCAGTGTTTCTCTTTGGGGAATCCTGGGATGGCTCTAGCCGTTCCGCAGACGGGATCGATTTCATGATTTTTTTTGTTTCGTTGCATAGGGTTTGGTTTGCCCTTTTCCTTTATTTCAATATATGCCGTGCACTTGTTTGTCGGGTCATCTTTTCATGCTTTTTTTTGTCTTGGTTGTGATGATGTGGTCTGGTTGGGCGGTCGTTCTAGATCGGAGTAGAATTAATTCTGTTTCAAACTACCTGGTGGATTTATTAATTTTGGATCTGTATGTGTGTGCCATACATATTCATAGTTACGAATTGAAGATGATGGATGGAAATATCGATCTAGGATAGGTATACATGTTGATGCGGGTTTTACTGATGCATATACAGAGATGCTTTTTGTTCGCTTGGTTGTGATGATGTGGTGTGGTTGGGCGGTCGTTCATTCGTTCTAGATCGGAGTAGAATACTGTTTCAAACTACCTGGTGTATTTATTAATTTTGGAACTGTATGTGTGTGTCATACATCTTCATAGTTACGAGTTTAAGATGGATGGAAATATCGATCTAGGATAGGTATACATGTTGATGTGGGTTTTACTGATGCATATACATGATGGCATATGCAGCATCTATTCATATGCTCTAACCTTGAGTACCTATCTATTATAATAAACAAGTATGTTTTATAATTATTTTGATCTTGATATACTTGGATGATGGCATATGCAGCAGCTATATGTGGATTTTTTTAGCCCTGCCTTCATACGCTATTTATTTGCTTGGTACTGTTTCTTTTGTCGATGCTCACCCTGTTGTTTGGTGTTACTTCTGCAGGTCGACTCTAGAGGATCCCCTCGAGGCGCGCCAAGCTATCAAACAAGTTTGTACAAAAAAGCAGGCTCCGAATTCGCCCTTCACCATGGCTCCTAAGAAGAAGCGGAAGGTTGGTATTCACGGGGTGCCTGCGGCTTCAAAGCTCGAGAAATTCACCAACTGTTATTCGTTGAGCAAAACACTGCGGTTTAAAGCGATTCCAGTCGGCAAGACTCAAGAGAATATAGACAATAAGCGGCTGTTGGTGGAAGATGAAAAGCGCGCGGAAGACTACAAAGGGGTGAAGAAGTTGTTGGACAGATACTACCTCTCTTTTATCAATGATGTCTTGCACTCAATCAAATTGAAGAATCTGAACAACTACATCTCCCTCTTCAGAAAGAAAACAAGGACAGAAAAGGAGAATAAGGAACTTGAAAATTTGGAGATCAATCTGAGGAAAGAGATCGCGAAAGCCTTTAAAGGCAACGAAGGATACAAAAGTCTGTTCAAGAAGGATATAATTGAGACAATTTTGCCAGAGTTCCTCGATGACAAGGACGAGATTGCGCTGGTCAATTCGTTCAACGGATTCACAACAGCATTCACAGGCTTCTTTGATAATCGGGAAAATATGTTCTCTGAGGAGGCAAAGTCCACTTCTATTGCGTTCAGGTGTATCAATGAGAATCTCACTAGGTACATTTCCAACATGGATATCTTTGAGAAGGTTGACGCAATTTTTGACAAGCACGAAGTTCAGGAGATTAAGGAGAAGATCCTCAATTCCGATTATGACGTTGAGGACTTCTTCGAAGGTGAGTTTTTTAATTTCGTGCTCACTCAAGAGGGTATCGACGTGTATAATGCGATCATCGGTGGGTTCGTGACTGAGTCCGGTGAAAAGATTAAGGGATTGAACGAGTATATCAACCTTTACAACCAAAAGACGAAACAGAAGCTGCCAAAGTTCAAGCCTCTTTACAAACAGGTTCTTTCAGACCGCGAGTCACTCTCGTTCTATGGGGAGGGCTACACTTCGGATGAGGAAGTCCTGGAGGTGTTCAGGAATACTCTCAATAAGAATTCGGAGATTTTCTCTTCTATAAAAAAACTGGAAAAGTTGTTTAAGAATTTTGACGAATACTCTAGCGCCGGCATATTTGTGAAAAACGGCCCGGCCATATCAACGATAAGTAAAGATATCTTCGGCGAATGGAACGTGATCAGAGACAAATGGAACGCGGAGTATGACGATATTCACCTGAAGAAGAAGGCTGTCGTAACGGAGAAGTACGAGGATGATCGCAGGAAAAGCTTCAAAAAGATCGGAAGTTTCAGCCTGGAACAGTTGCAGGATCGTTCAAACATGAGTATGCTGACGCCGATCTTAGCGTCGTCGAGAAGTTGAAGGAGATAATCATCCAAAAGGTCGACGAGATATATAAAGTCTATGGATCAAGTGAAAAACTGTTCGACGCCGACTTCGTTTTGGAGAAGTCCCTGAAGAAGAACGACGCTGTTGTTGCCATTATGAAGGATCTGCTCGACAGCGTGAAGAGTTTCGAGAACTATATTAAGGCTTTTTTCGGGGAGGGGAAGGAGACTAACAGAGATGAGTCCTTCTACGGAGACTTCGTCCTCGCGTACGATATACTCCTTAAGGTAGACCACATCTACGACGCAATCAGAAATTACGTGACACAAAAGCCGTACAGCAAGGACAAGTTCAAACTCTACTTCCAGAACCCCCAGTTCATGGGCGGCTGGGACAAGGACAAGGAAACGGATTACAGGGCTACGATCCTGAGGTATGGTTCAAAATACTACTTGGCGATTATGGACAAGAAGTACGCCAAGTGTCTCCAGAAGATTGACAAAGACGATGTCAATGGCAATTATGAGAAGATCAACTACAAGCTGCTTCCGGGTCCGAACAAGATGCTCCCAAAGGTTTTCTTCAGCAAGAAATGGATGGCCTACTATAACCCAAGCGAGGACATCCAGAAGATTTATAAGAACGGTACGTTCAAGAAGGGCGACATGTTCAATCTTAACGACTGTCACAAGCTGATCGACTTCTTCAAAGACTCAATTAGCCGGTACCCAAAGTGGTCTAACGCCTATGACTTCAACTTTTCGGAAACCGAGAAGTACAAGGATATAGCCGGATTTTATAGAGAGGTGGAAGAGCAGGGCTACAAGGTGTCATTCGAGTCCGCCAGCAAGAAGGAAGTGGACAAGCTCGTGGAAGAGGGTAAGCTCTACATGTTCCAGATTTATAATAAAGACTTTAGCGATAAGAGCCACGGGACACCTAATCTCCACACAATGTATTTCAAGCTGCTCTTCGACGAGAATAACCACGGCCAAATCAGGTTGTCAGGAGGGGCTGAACTCTTCATGCGGCGCGCTAGCCTTAAGAAGGAGGAGCTTGTAGTCCACCCTGCGAATAGTCCAATTGCGAATAAGAACCCGGACAATCCTAAAAAGACTACAACATTGAGCTACGACGTGTACAAGGATAAGAGGTTTTCCGAGGATCAGTACGAGCTCCACATCCCGATTGCGATCAACAAGTGCCCAAAGAATATTTTCAAGATAAACACAGAGGTGCGTGTACTCCTGAAGCATGACGACAATCCTTACGTCATTGGGATTGATCGGGGCGAGAGGAACCTCCTCTATATTGTGGTGGTGGACGGGAAGGGGAACATAGTCGAACAGTACTCCCTTAACGAAATAATTAACAATTTCAACGGCATCCGTATCAAGACCGACTACCATTCGTTGCTGGACAAGAAGGAGAAGGAGAGATTTGAGGCGCGGCAAAATTGGACAAGTATCGAGAACATCAAGGAACTCAAAGCAGGTTATATCTCTCAAGTTGTGCATAAGATATGCGAGCTGGTTGAGAAGTATGACGCAGTGATCGCTCTTGAGGACCTCAACTCGGGCTTTAAGAATTCTAGAGTTAAAGTGGAGAAGCAGGTCTATCAAAAGTTCGAGAAGATGCTTATAGATAAGCTCAACTACATGGTCGATAAGAAATCGAACCCATGTGCCACCGGCGGCGCACTCAAAGGTTACCAAATAACAAACAAATTCGAGTCCTTCAAATCGATGAGTACTCAGAATGGGTTCATATTTTATATACCGGCGTGGCTTACGTCTAAGATCGACCCGTCAACTGGTTTTGTCAACCTGTTGAAGACGAAATACACGTCCATTGCCGATTCGAAAAAGTTCATATCTAGTTTTGATCGTATTATGTACGTCCCAGAGGAAGATCTTTTCGAGTTTGCTCTCGACTACAAAAACTTTTCGCGGACCGATGCGGATTACATTAAAAAATGGAAACTCTATTCGTACGGCAACAGAATCAGGATTTTTCGCAACCCTAAGAAGAATAACGTCTTTGATTGGGAGGAAGTTTGCTTGACTAGCGCGTACAAGGAGCTCTTTAATAAGTATGGCATTAACTACCAACAGGGTGATATCAGAGCACTGCTTTGCGAACAATCTGACAAGGCTTTCTACTCATCCTTCATGGCTTTGATGAGCCTGATGCTCCAGATGAGAAATTCAATTACAGGCAGAACCGACGTGGATTTCTTGATCTCCCCGGTTAAAAATTCTGATGGCATCTTTTACGATAGCAGGAACTATGAAGCGCAAGAGAATGCGATTCTGCCAAAAAATGCAGACGCCAACGGTGCCTATAACATCGCCAGGAAAGTCCTGTGGGCGATCGGCCAGTTCAAAAAGGCCGAAGACGAAAAATTGGACAAGGTCAAAATCGCTATCAGCAACAAAGAGTGGCTGGAGTATGCTCAGACATCCGTAAAGCATAAGCGTCCTGCTGCCACCAAAAAGGCCGGACAGGCTAAGAAAAAGAAGTGAGACGACTAGACCCAGCTTTCTTGTACAAAGTGGTTGATAACAGCGACTACAAGGATGACGATGACAAGGCTTAGAGCTCGAATTTCCCCGATCGTTCAAACATTTGGCAATAAAGTTTCTTAAGATTGAATCCTGTTGCCGGTCTTGCGATGATTATCATATAATTTCTGTTGAATTACGTTAAGCATGTAATAATTAACATGTAATGCATGACGTTATTTATGAGATGGGTTTTTATGATTAGAGTCCCGCAATTATACATTTAATACGCGATAGAAAACAAAATATAGCGCGCAAACTAGGATAAATTATCGCGCGCGGTGTCATCTATGTTACTAGATC

**Supplementary Figure S7. Structure and essential DNA sequence features of plasmids pSH924A (a) and pSH984 (b), used for CRISPR/Cpf1–mediated knockout of the rice Pho1 gene.** The *PHO1* guide sequence was inserted into the *Bsa*I-*Bsa*I gap of the guide RNA (gRNA) expression cassette in pSH924A, which contains the rice U6B2 promoter, direct repeat, cloning site, and terminator. The resulting plasmid pSH984T was amplified by PCR and cloned into the *Bsa*I sites of the backbone vector pSH916 (derived from pYPQ230 and pRGEB31), yielding pSH984. DNA sequence features are highlighted in the following order: (a) green, rice small nuclear RNA U6B2 promoter; light gray, direct repeat; magenta, guide sequence cloning site; black, RNA polymerase III terminator; dark gray, 3′ region of the rice U6B2 gene. (b) green, rice U6B2 promoter; light gray, direct repeat; red, guide (target) sequence in exon-2 of rice *PHO1*; black, RNA polymerase III terminator; dark gray, 3′ region of the rice U6B2 gene; cyan, maize ubiquitin promoter; yellow, *Lb*Cpf1; and dark gray, *nos* terminator.
