# Supplementary material for: Starch phosphorylase acts as a pivotal scaffold connecting starch metabolism enzymes with photosystem I subunits
Source: Plant Cell Physiol. 2026 May 14;67(8):1430–41. doi: 10.1093/pcp/pcag063 (PMC13545646; doi:10.1093/pcp/pcag063)
Supplement: Supplementary file 1 [file supplementary_material_pcag063.zip › Supplementary Figure S3.docx]

**Supplementary Figure S3.** **Yeast two-hybrid assay of protein-protein interactions between the L80 region of Pho1 and PS-I subunits.** Yeast transformants (1 to 20) co-expressing bait proteins (PsaC, HT-PsaC, PsaD, and PsaE) fused to the GAL4 DNA-binding domain (GBD) and prey proteins (HT, HT:L80, HT:L80E, L80, and L80E) fused to the GAL4 activation domain (GAD) were analyzed for protein-protein interactions. Transformants (a to t) represent the reciprocal vector arrangements. Cells were streaked onto selective synthetic dropout (SD) media: SD/-2 (left plates) and SD/-4 (right plates) to assess growth. Empty vectors (pGBKT7.1 and pGADT7.1) served as negative controls, while constructs
