# Supplementary material for: Starch phosphorylase acts as a pivotal scaffold connecting starch metabolism enzymes with photosystem I subunits
Source: Plant Cell Physiol. 2026 May 14;67(8):1430–41. doi: 10.1093/pcp/pcag063 (PMC13545646; doi:10.1093/pcp/pcag063)
Supplement: Supplementary file 1 [file supplementary_material_pcag063.zip › Supplementary Figure S4.docx]

**Supplementary Figure S4.** **Yeast two-hybrid assay analyzing protein-protein interactions between PS-I subunits with or without HaloTag (HT).** Bait proteins (left) were fused to the GAL4 DNA-binding domain (GBD), and prey protein (right) were fused to the GAL4 activation domain (GAD). Yeast cells co-transformed with GBD-bait constructs, HT-PsaC (a), HT-PsaD (b), and HT-PsaE (c) and GAD-prey constructs (PasC, PsaD, and PsaE, with or without HT) were streaked onto selective synthetic dropout (SD) media: SD/-2 and SD/-4. Growth on SD/-4 indicates interaction-dependent activation of reporter genes. See Materials and Methods and the legend of Figure 3 for further details.
