# Supplementary material for: Starch phosphorylase acts as a pivotal scaffold connecting starch metabolism enzymes with photosystem I subunits
Source: Plant Cell Physiol. 2026 May 14;67(8):1430–41. doi: 10.1093/pcp/pcag063 (PMC13545646; doi:10.1093/pcp/pcag063)
Supplement: Supplementary file 1 [file supplementary_material_pcag063.zip › Supplementary Figure S5.docx]

**Supplementary Figure S5. Heterologous expression and purification of 6His-HaloTag–PsaC**. Proteins were resolved by 12.5% SDS-polyacrylamide gel electrophoresis (Laemmli, 1970) and stained with Coomassie Brilliant Blue G-250. (a) Protein production was induced with 0.1 M IPTG in *E. coli* EA3457 cells. Soluble fractions were obtained after cell lysis using lysozyme and sonication. (b) The soluble proteins were applied to TALON immobilized metal affinity chromatography (IMAC; GE Healthcare), and bound proteins were eluted with 250 mM imidazole.
