# Supplementary material for: Starch phosphorylase acts as a pivotal scaffold connecting starch metabolism enzymes with photosystem I subunits
Source: Plant Cell Physiol. 2026 May 14;67(8):1430–41. doi: 10.1093/pcp/pcag063 (PMC13545646; doi:10.1093/pcp/pcag063)
Supplement: Supplementary file 1 [file supplementary_material_pcag063.zip › Supplementary Table S1.docx]

**Supplementary Table S1. Analysis of interactions between Pho1 subunits from wildtype rice and BMF136 mutant.** The YASARA software was used to quantify total contacts, hydrogen bonds, and hydrophobic interactions between the two subunits. The Pho1 protein in BMF136 contains a 53-amino acid deletion located at the interface between the two subunits:

* Hydrogen bonds with energies greater than 6.25 kJ/mol were counted. Nine hydrogen bonds were detected in total, with one subunit contributing five and the other contributing four.

>Truncated Pho1 in BMF136 MATASAPLQLATASRPLPVGVGCGGGGGGGLHVGGARGGGAAPARRRLAVRSVASDRGVQGSVSPEEEISSVLNSIDSSTIASNIKHHAEFTPVFSPEHFSPLKAYHATAKSVLDTLIMNWNATYDYYDRTNVKQAYYLSMEFLQGRALTNAVGNLELTGQYAEALQQLGHSLEDVATQEPDAALGNGGLGRLASCFLDSLATLNYPAWGYGLRYKHGLFKQIITKDGQEEVAENWLEMGNPWEIVRTDVSYPVKFYGKVVEGTDGRMHWIGGENIK[-----------------------------------------------------]ICHVLYPGDESPEGKVLRLKQQYTLCSASLQDIIARFERRAGDSLS WEDFPSKVAVQMNDTHPTLCIPELMRILIDVKGLSWNEAWSITERTVAYTNHTVLPEALEKWSLDIMQKLLPRHVEIIEKIDGELMNIIISKYGTEDTSLLKKKIKEMRILDNIDLPDSIAKLFVKPKEKKESPAKLKEKLLVKSLEPSVVVEEKTVSKVEINEDSEEVEVDSEEVVEAENEDSEDELDPFVKSDPKLPRVVRMANLCVVGGHSVNGVAAIHSEIVKEDVFNSFYEMWPAKFQNKTNGVTPRRWIRFCNPELSAIISKWIGSDDWVLNTDKLAELKKFADDEDLQSEWRAAKKANKVKVVSLIREKTGYIVSPDAMFDVQVKRIHEYKRQLLNILGIVYRYKKMKEMSAKDRINSFVPRVCIFGGKAFATYVQAKRIVKFITDVAATVNHDPEIGDLLKVVFIPDYNVSVAEALIPASELSQHISTAGMEASGTSNMKFAMNGCILIGTLDGANVEIREEVGEENFFLFGAEAHEIAGLRKERAQGKFVPDPRFEEVKRFVRSGVFGTYNYDDLMGSLEGNEGYGRADYFLVGKDFPSYIECQEKVDKAYRDQKLWTRMSILNTASSSKFNSDRTIHEYAKDIWDIKPVILP
