# Supplementary material for: Starch phosphorylase acts as a pivotal scaffold connecting starch metabolism enzymes with photosystem I subunits
Source: Plant Cell Physiol. 2026 May 14;67(8):1430–41. doi: 10.1093/pcp/pcag063 (PMC13545646; doi:10.1093/pcp/pcag063)
Supplement: Supplementary file 1 [file supplementary_material_pcag063.zip › Supplementary Table S2.docx]

**Supplementary Table S2. Oligonucleotide primers used in this study.**

| **Primer** | **Sequence (5' to 3')** | **Purpose** |
| --- | --- | --- |
| PHO1-cR1-F | AGATACCTCTGAAGGCTTACCATGCAA | Forward primer for guide sequence at exon-2 in rice Pho1 (CRISPR) |
| PHO1-cR1-R | TGGAGACTTCCGAATGGTACGTTAAAA | Reverse primer for guide sequence at exon-2 in rice Pho1 (CRISPR) |
| PHO1_ex2-F | TGTAATGCAGAGATTTCAAGTGTGCT | Amplification of Pho1 exon-2 (CRISPR) |
| PHO1_ex2-R | TAGCAACATCCTCTAGGCTGTGTC | Amplification of Pho1 exon-2 (CRISPR) |
| PHO1_ex2-seqF | CCATCGATTCCTCTACCATTGCAT | Sequencing primer for Pho1 exon-2 (CRISPR) |
| U3-BsaF | TTAGGGTCTCTGGCATACGAACAGATCACTTAAAGTTCTTCT | cloning of rice U3 promoter & terminator (CRISPR) |
| U3t-BsaR | CTTGGTCTCCAAACGCTGTGCCGTACGACGGTA | cloning of rice U3 promoter & terminator (CRISPR) |
| U6B2-BsaF | AAGGTCTCTGGACCGATCTGCCGCCGGATCATG | cloning of rice U6B promoter & terminator (CRISPR) |
| U6B2t-BsaR | AGAGGTCTCAAAACTGACCATTTGTGGGTCTGT | cloning of rice U6B promoter & terminator (CRISPR) |
| L80-H3F | CTGAAGCTTTGGAGAAGTGGAGCTTGGAC | cloning of L80 region from Pho1 cDNA |
| L80-EcR | ACCGAATTCAACGTCTAGGAGTCACTCCAT | cloning of L80 region from Pho1 cDNA |
| L80seq-F | GCAGAAACTTCTTCCTCGGCATGT | cloning of L80 region from Pho1 cDNA |
| L80seq-R | TTGAAATTTAGCGGGCCACATCTC | cloning of L80 region from Pho1 cDNA |
| L80seq-R2 | TTTGAAATTTAGCGGGCCACATCT | cloning of L80 region from Pho1 cDNA |
| L80-H3F2 | CTGAAGCTTTGGAGAAGTGGAGCTTGGAC | cloning of L80 region from Pho1 cDNA |
| L80-EcR2 | ACCGAATTCAACGTCTAGGAGTCACTCCAT | cloning of L80 region from Pho1 cDNA |
| L80seq-F2 | GCAGAAACTTCTTCCTCGGCATGT | cloning of L80 region from Pho1 cDNA |
| L80SEc-F | TTAGAATTCATTGACCTACCAGATTCTATTGC | cloning of L80 region from Pho1 cDNA |
| L80LEc-F | GATGAATTCCTGATGAACATCATTATCTCAA | cloning of L80 region from Pho1 cDNA |
| L80SPst-R | TTTCTGCAGTTATTTTACAAATGGATCTAACTCATC | cloning of L80 region from Pho1 cDNA |
| L80LPst-R | TCGCTGCAGTTAAGGTAATTTAGGATCTGATTTTAC | cloning of L80 region from Pho1 cDNA |
| BMFPho1F2 | GTTGTTGCTCATGATATCCCTATTCCTG | Cloning of Pho1 cDNA from rice BMF136 |
| BMFPho1F | GTCAACAACAGTGCCATCACAAG | Cloning of Pho1 cDNA from rice BMF136 |
| BMFPho1R | ACAGTGATGGCAATGAGCAATACTAG | Cloning of Pho1 cDNA from rice BMF136 |
| 1192-Xho-R | CCCCTCGAGACCCGTTTAGAGGCCCCA | Cloning of L80 region |
| L-K823A-F | AGTGGAACCAGCAACATGGCTTTTGCAATGAATGGATGT | mutagenesis of amino acid residue for PLP binding (Pho1 and Pho2) |
| L-K823A-R | ACATCCATTCATTGCAAAAGCCATGTTGCTGGTTCCACT | mutagenesis of amino acid residue for PLP binding (Pho1 and Pho2) |
| 35ST-R | TCTGGGAACTACTCACACATTATTCTG | Sequencing primer for pSH1122N pSH1123C |
| YFPPseq-F | GGAGAGGACGTCGAGAGTTCTCAA | Sequencing primer for pSH1122N pSH1123C |
| NYFPseq-F | AGTACAACTACAACAGCCACAACGT | Sequencing primer for pSH1122N |
| CYFPseq-F | CGATCACATGGTCCTGCTGGAGTTC | Sequencing primer for pSH1123C |
| GADT7-seqF | CTATCTATTCGATGATGAAGATACC | Sequencing primer for pGADT7.1 |
| GADT7-seqF2 | GATCTTTAATACGACTCACTATAGG | Sequencing primer for pGADT7.1 |
| GADT7-seqR | AGATGGTGCACGATGCACAG | Sequencing primer for pGADT7.1 |
| GBKT7-seqF | CAGCATAGAATAAGTGCGACATCA | Sequencing primer for pGBKT7.1 |
| GBKT7-seqR | CGGAATTAGCTTGGCTGCAAG | Sequencing primer for pGBKT7.1 |
| PsaD-NcF1 | ACCATGGCCATGGCCACGCAAGCCTCCG | Cloning of rice PsaD |
| PsaD-NcF2 | AGCCCATGGCAGCAGCGACGGAGGAGAAGAA | Cloning of rice PsaD |
| PsaD-H3R | AGAAGCTTAGATGTCGAAGACGTTCTTGC | Cloning of rice PsaD |
| PsaE-NcF | CGGCCATGGCGAGCACCATTAACGTGGCGTC | Cloning of rice PsaE |
| PsaE-H3R | AATAAGCTTCATTTGACCTCCTGGATCTCGTCCA | Cloning of rice PsaE |
| PsaEint1-F | AAGATCCTGAGGAGGGAGTCCTACTG | Cloning of rice PsaE (removal of intron) |
| PsaEint1-R | CACCTTGGTGCCTCTCTTGGGGCCAAT | Cloning of rice PsaE (removal of intron) |
| PsaEint2-F | GATCCCAACACTCGCTACCCGGTGGT | Cloning of rice PsaE (removal of intron) |
| PsaEint2-R | CTGATCAACGGTGACGACGGAGCCAG | Cloning of rice PsaE (removal of intron) |
