# Supplementary material for: Starch phosphorylase acts as a pivotal scaffold connecting starch metabolism enzymes with photosystem I subunits
Source: Plant Cell Physiol. 2026 May 14;67(8):1430–41. doi: 10.1093/pcp/pcag063 (PMC13545646; doi:10.1093/pcp/pcag063)
Supplement: Supplementary file 1 [file supplementary_material_pcag063.zip › Supplementary Table S3.docx]

**Supplementary Table S3. Overview of plasmid construction**

| **Protein** | **Final construct** | **Destination Vector (to)** | **Cloning sites** | **Source DNA (from)** | **Cloning method** |
| --- | --- | --- | --- | --- | --- |
| nEYFP::Pho1 | pSH1135 | pSAT1-nEYFP-C1 | SalI/Kpn | pSH1125H | SalI/KpnI digestion & ligation |
| cEYFP::PsaC | pSH1141 | pSAT1-cEYFP-C1-B | BglII/HindIII | pSH993 | BamHI/HindIII digestion & ligation |
| cEYFP::Pho1 | pSH1155 | pSAT1-cEYFP-C1-B | SmaI/SalI | pSH1135 | Smai/SalI digestion & ligation |
| PHO1 cRNA on pSH923B | pSH983T | pSH923B | BsaI | PHO1-cR1-F and PHO1-cR1-R2 | annealed |
| PHO1 cRNA on pSH924A | pSH984T | pSH924A | BsaI | PHO1-cR1-F and PHO1-cR1-R2 | annealed |
| U3::DR::PHO1-cR1::Term | pSH983 | pSH916 | BsaI | PCR product (gRNA expression cassette) of pSH983T (w/U3-BsaF/U3t-BsaR) | BsaI digestion & ligation |
| U6B2::DR::PHO1-cR1::Term | pSH984 | pSH916 | BsaI | PCR product (gRNA expression cassette) of pSH984T (w/U6B2-BsaF/U6B2t-BsaR) | BsaI digestion & ligation |
| GBD:Pho1 | pSH997 | pGBKT7.1 | NcoI/KpnI | pSH585 | NcoI/KpnI digestion & ligation |
| GBD:Pho1∆L80 | pSH1000 | pGBKT7.1 | NcoI/KpnI | pSH996 | NcoI/KpnI digestion & ligation |
| GAD:Pho1∆L80 | pSH1001 | pGADT7.1 | NcoI/SalI | pSH996 | NcoI/SalI digestion & ligation |
| GAD:Pho1 | pSH1002 | pGADT7.1 | NcoI/SalI | pSH997 | NcoI/SalI digestion & ligation |
| GBD:PsaC | pSH1003 | pGBKT7.1 | NcoI/SalI | pSH993 | NcoI/SalI digestion & ligation |
| GAD:PsaC | pSH1004 | pGADT7.1 | NcoI/SalI | pSH1003 | NcoI/SalI digestion & ligation |
| GBD:PsaD (mature) | pSH1005 | pGBKT7.1 | NcoI/SalI | pSH995 | NcoI/SalI digestion & ligation |
| GAD:PsaD (mature) | pSH1006 | pGADT7.1 | NcoI/SalI | pSH1005 | NcoI/SalI digestion & ligation |
| GBD:Pho1[K823A] | pSH1007 | pGBKT7.1 | NcoI/KpnI | pSH998 | NcoI/KpnI digestion & ligation |
| GBD:Pho1∆L80[K823A] | pSH1008 | pGBKT7.1 | NcoI/KpnI | pSH999 | NcoI/KpnI digestion & ligation |
| GAD:Pho1[K823A] | pSH1009 | pGADT7.1 | NcoI/SalI | pSH998 | NcoI/SalI digestion & ligation |
| GAD:Pho1∆L80[K823A] | pSH1010 | pGADT7.1 | NcoI/SalI | pSH999 | NcoI/SalI digestion & ligation |
| GBD::mature PsaE | pSH1047 | pGBKT7.1 | NcoI/SacI | pSH1046T | NcoI/SacI digestion & ligation |
| GAD::mature PsaE | pSH1049 | pGADT7.1 | NcoI/EcoRI | pSH1047 | NcoI/EcoRI digestion & ligation |
| GAD:HT:PsaC | pSH1108 | pSH1043 | BamHI/SacI | pSH1107 | BamHI(partial)/SacI digestion & ligation |
| GBD:HT:PsaC | pSH1109 | pSH1042 | BamHI/KpnI | pSH1108 | BamHI/KpnI digestion & ligation |
| GBD::OsDpe1 | pSH1167 | pGBK685NE | EcoRI/SalI | pSH685 | EcoRI/SalI digestion & ligation |
| GAD::OsDpe1 | pSH1168 | pGAD_685NE | EcoRI/XhoI | pSH685 | EcoRI/SalI digestion & ligation |
| GAD:HT | pSH1191 | pGADT7.1 |  | pSH1108 | SalI-SalI fragment (PsaC) was deleted |
| GBD:HT | pSH1192 | pGBKT7.1 | NcoI/SalI | pSH1108 | NcoI/XhoI digestion & ligation |
| GBD:HT | pSH1192.1 | pGBKT7.1 | NcoI/SalI | pSH1108 | NcoI/XhoI digestion & ligation. Multiple restriction sites remained after ligation. |
| GBD:HT:L80 | pSH1193 | pSH1192.1 | EcoRI/PstI | PCR product (L80SEc-F/L80SPst-R) of pSH407 | EcoRI/PstI digestion & ligation |
| GBD:HT:L80 extended | pSH1194 | pSH1192.1 | EcoRI/PstI | PCR product (L80LEc-F/L80LPst-R) of pSH407 | EcoRI/PstI digestion & ligation |
| GBD:L80 | pSH1195 |  |  | pSH1193 | BamHI-BamHI region was deleted |
| GBD:L80 extended | pSH1196 |  |  | pSH1194 | BamHI-BamHI region was deleted |
| GAD:HT:L80 | pSH1197 | pGADT7.1 | NcoI/XhoI | PCR product (GBKT7seq-f/1192-XhoI-R) of pSH1193 | NcoI/XhoI digestion & ligation |
| GAD:HT:L80 entended | pSH1198 | pGADT7.1 | NcoI/XhoI | PCR product (GBKT7seq-f/1192-XhoI-R) of pSH1194 | NcoI/XhoI digestion & ligation |
| GAD:L80 | pSH1199 |  |  | pSH1197 | BamHI-BamHI region was deleted |
| GAD:L80 extended | pSH1200 |  |  | pSH1198 | BamHI-BamHI region was deleted |
| GBD:HT:PsaC | pSH1299 | pSH1109 | BamHI/KpnI | pSH1296 | BamHI(partial)/KpnI digestion & ligation |
| GBD:HT:PsaD | pSH1300 | pSH1109 | BamHI/SmaI | pSH1297 | BamHI/HindIII (blunted) digestion & ligation |
| GBD:HT:PsaE | pSH1301 | pSH1109 | BamHI/PstI | pSH1301T | BamHI(partial)/PstI digestion & ligation |
| GAD:HT:PsaD | pSH1308 | pGADT7.1 | NdeI/SmaI | pSH1300 | NdeI+PstI (blunted) digestion & ligation |
| GAD:HT:PsaE | pSH1309 | pGADT7.1 | NdeI/SmaI | pSH1301 | NdeI+PstI (blunted) digestion & ligation |
| GST:Factor Xa:GFP | pSH1440 | pGEX-2T | BamHI+EcoR | pSH659Td | BamHI/EcoR digestion & ligation |
| GST:Factor Xa:DPE1 | pSH1441 | pSH1440 | BamHI-NheI | pSH670 | BamHI/NheI digestion & ligation |
| GST:Factor Xa:Pho1 | pSH1442 | pSH1440 | NcoI-SalI | pSH1002 | NcoI/SalI digestion & ligation |
| GST:Factor Xa:Pho1∆L80 | pSH1443 | pSH1440 | NcoI-SalI | pSH1000 | NcoI/SalI digestion & ligation |
| rice PsaD full-length | pSH969 | pSH966 | NcoI/HindIII | PCR product of PsaD with PsaD-NcF1/PsaD-H3R on AK120372 | NcoI/HindIII digestion & ligation |
| mature rice PsaD | pSH970 | pSH966 | NcoI/HindIII | PCR product of PsaD with PsaD-NcF2/PsaD-H3R on AK120372 | NcoI/HindIII digestion & ligation |
| 6His::PsaC | pSH993 | pSH967 | NcoI/KpnI | pSH948 (pSH952) | NcoI/KpnI digestion & ligation |
| 6His::PsaC | pSH993xHK |  |  | pSH993 | HindIII/KpnI, blunted, and self-ligated |
| PsaE gDNA | pSH994 | pSH966 | NcoI/HindIII | PCR product from rice Kitaake gDNA with PsaE-NcF/ PsaE-H3R | NcoI/HindIII digestion & ligation |
| 6His::PsaE | pSH994D |  |  | 1st: PCR product of pSH994 with PsaEint1-F/PsaEint1-R. 2nd: PCR product of the pooled pDNAs with PsaEint2-F/PsaEint2-R | Self-ligation to remove an intron |
| 6His::PsaE | pSH994D-8 | pSH280.1. | NcoI/HindIII | pSH994D | NcoI/HindIII digestion & ligation |
| 6His::PsaD (matuire) | pSH995 | pSH967B | NcoI/HindIII | pSH970 | NcoI/HindIII digestion & ligation |
| 6His::PsaD (matuire) | pSH995xHK |  | HindIII/KpnI | pSH995 | HindIII/KpnI, blunted, and self-ligated |
| 6His:Pho1[K823A] | pSH537 | pSH533 | BamHI/KpnI | pSH508 (K823A was introduced into OsPho1 by site-directed mutagenesis) | BamHI/KpnI digestion & ligation |
| 6His:HT:Pho1[K823A] | pSH999 | pSH996 | BglII/KpnI | pSH537 | BglII/KpnI digestion & ligation |
| 6His:Pho1∆L80 [K823A] | pSH1113K | pSH967NK | NcoI/SalI | pSH999 | NcoI/SalI digestion & ligation |
| 6HIs:HT:PsaC | pSH1107 | pSH967NK | EcoRI/HindIII | pKK32 | BamHI/HIndIII digestion & ligation |
| 6HIs:HT:PsaD | pSH1318 | pSH1295GH | NcoI/SacI | pSH1308 | NcoI(partial)/SacI digestion & ligation |
| 6HIs:HT:PsaE | pSH1319 | pSH1295GH | NcoI/SacI | pSH1309 | NcoI(partial)/SacI digestion & ligation |
